# Numerical modelling of label-structured cell population growth using CFSE distribution data

**DOI:** 10.1186/1742-4682-4-26

**Published:** 2007-07-24

**Authors:** Tatyana Luzyanina, Dirk Roose, Tim Schenkel, Martina Sester, Stephan Ehl, Andreas Meyerhans, Gennady Bocharov

**Affiliations:** 1 Institute of Mathematical Problems in Biology, RAS, Pushchino, Russia; 2 Department of Computer Science, Katholieke Universiteit Leuven, Belgium; 3 Department of Virology, University of the Saarland, Homburg, Germany; 4 Department of Internal Medicine, University of the Saarland, Homburg, Germany; 5 Children's Hospital, University of Freiburg, Freiburg, Germany; 6 Institute of Numerical Mathematics, RAS, Moscow, Russia

## Abstract

**Background:**

The flow cytometry analysis of CFSE-labelled cells is currently one of the most informative experimental techniques for studying cell proliferation in immunology. The quantitative interpretation and understanding of such heterogenous cell population data requires the development of distributed parameter mathematical models and computational techniques for data assimilation.

**Methods and Results:**

The mathematical modelling of label-structured cell population dynamics leads to a hyperbolic partial differential equation in one space variable. The model contains fundamental parameters of cell turnover and label dilution that need to be estimated from the flow cytometry data on the kinetics of the CFSE label distribution. To this end a maximum likelihood approach is used. The Lax-Wendroff method is used to solve the corresponding initial-boundary value problem for the model equation. By fitting two original experimental data sets with the model we show its biological consistency and potential for quantitative characterization of the cell division and death rates, treated as continuous functions of the CFSE expression level.

**Conclusion:**

Once the initial distribution of the proliferating cell population with respect to the CFSE intensity is given, the distributed parameter modelling allows one to work directly with the histograms of the CFSE fluorescence without the need to specify the marker ranges. The label-structured model and the elaborated computational approach establish a quantitative basis for more informative interpretation of the flow cytometry CFSE systems.

## Background

Understanding the dynamics of cell proliferation, differentiation and death is one of the central problems in immunology [1]. A cell population is an ensemble of individual cells, all of which contribute in a different way to the overall observed behavior [2]. A quantitative characterization of this heterogeneity is provided by flow cytometry. Flow cytometry is a technique based on the use of fluorescence activated cell sorter (FACS) for a quantitative single cell analysis of the suspensions of cells, which are labelled with fluorescent substance(s). Once the labelled cells are run through the cell sorter machine, the computer collects data on the fluorescence intensity for each cell [3]. The FACS is capable of analyzing up to a dozen parameters per cell at rates up to 10^5 ^cells per second. Therefore it represents a versatile tool with an enormous potential to describe the complex nature of cell populations [4].

**Figure 1:**
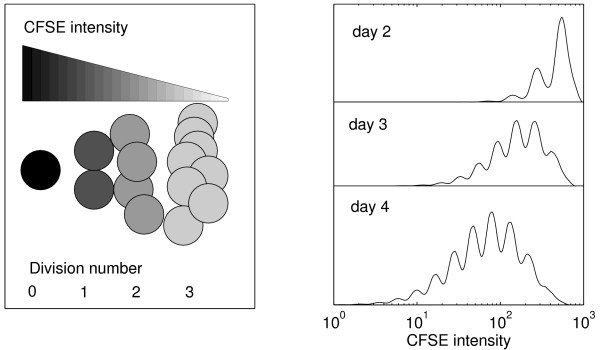
CFSE dilution (left) and typical CFSE intensity histograms (right).

Various labelling techniques are available for the analysis of the lymphocyte proliferation in response to stimuli indicing cell division. These include, for example, carboxy-fluorescein diacetate succinimidyl ester (CFSE) labelling, the use of bromodeoxyuridine (BrdU) which incorporates into the DNA of dividing cells, ^3^H thymidine incorporation analysis, the expression of the nuclear *Ki *– 67 antigen in the nuclei of cycling cells. The use of CFSE to track cell division gives several advantages over the other labelling assays [5,6]: the lack of radioactivity; no antibody required to detect CFSE; when using CFSE assay viable cells can be recovered for further phenotypic examination; it is possible to apply different initial staining for different cell subsets so that complex mixtures of cells can be analyzed. The major aspects of CFSE function can be summarized as follows: (*i*) CFSE consists of a fluorescein molecule containing a succinimidyl ester functional group and two acetate moieties; (*ii*) it diffuses freely into cells and intracellular esterases cleave the acetate groups converting them to a fluorescent, membrane impermanent dye; (*iii*) CFSE is retained by the cell in the cytoplasm and does not adversely affect cellular function; (*iv*) during each round of cell division, the fluorescent CFSE is partitioned equally between daughter cells, see Fig. 1 (left). The histograms of the CFSE intensity distribution for proliferating cell populations can be obtained by FACS at various times, cf. Fig. 1 (right), providing the raw data for further quantitative analysis of the kinetics of cell division. This method permits the identification of up to 10 successive cell generations [6,7].

A thorough interpretation and comprehensive understanding of CFSE-labelled lymphocytes population data requires both the development of quantitatively consistent mathematical models, e.g. based on distributed parameter systems such as hyperbolic partial differential equations, and efficient computational techniques for the solution and identification of these models. The heterogeneity of the dividing cell populations can be described by a wide range of characteristics, e.g. the number of divisions made, the position in the cell cycle, the mass, the label expression, the doubling time, the death rate. The mathematical modelling approaches for the analysis of cell growth from CFSE assay data developed so far consider the cell populations as a mixture of cells which differ only in the mean level of the CFSE expression per generation [7-11]. The cells within each generation (compartment) are assumed to possess the same constant level of CFSE fluorescence which is reduced by a factor of 2 after one division. Most of the models ignore the heterogeneity of cell populations with respect to the division and death rates, except for the naive versus dividing cells. The effect of cell heterogeneity with respect to the division times in the context of CFSE data analysis is explored in [8]. An extended comparative analysis of the existing compartmental models for CFSE-labelled cell growth has recently been presented in [12]. These models, formulated using ordinary or delay differential equations, consider the dynamics of the consecutive generations of dividing cells but not the single cell identity. Hence they can be referred to as unstructured and non-corpuscular, following the definitions in [13].

Distributed population balance models, which use partial differential equations (PDEs), are regarded as the most general way of describing heterogenous cell systems. Such models are considerably more difficult to analyze mathematically and numerically than their unstructured counterparts. The most extensively studied distributed parameter models for population dynamics are the age-structured models [14-16]. The only example of application of the age-maturity structured model for the CFSE data analysis is presented in [17]. The cell population is considered to be continuously structured with respect to the cell age, but the maturity variable (the CFSE fluorescence) is discrete, i.e., *k *distinct cell generations are considered, each characterized by some average CFSE fluorescence per cell, *M*/2^*k*^, with *M *the initial fluorescence. The division and death rates are assumed to be independent of the maturity and they are estimated by fitting experimental data with the model visually. In general, for cell growth problems the age-structured population models are considered to be of limited practical value due to the fact that the cell age is difficult to measure experimentally [13].

A class of distributed parameter models for cell populations growth, which allows direct reference to the experimentally measurable properties of cells, is represented by so-called size- or mass-structured cell populations models [4,18-20]. The terms ”size” and ”mass” refer to any cell property which satisfies a conservation law, e.g. volume, protein content, fluorescence label, etc. A rigorous mathematical analysis of such models was presented in [21]. The mass-structured population balance models are considered to provide a consistent way to estimate the fundamental physiological functions from flow cytometry data in the area of biotechnology [4,13].

In this study we formulate a one-dimensional first order hyperbolic PDE model for the dynamics of cell populations structured according to the CFSE fluorescence level. This structure variable defines the division age of the cell. We let the fluorescence intensity of the initial cell population and, therefore, of the consecutive generations to range continuously in some interval, thus relaxing a restricting assumption of an equal expression of CFSE by cells which have undergone the same number of divisions.

The proposed CFSE label-structured model potentially has the following advantages with respect to existing compartmental models: (*i*) it allows one to estimate the turnover parameters directly from the distributions of CFSE-labelled cells followed over time by flow cytometry; (*ii*) it does not require an ad hoc assumption on the relationship between the label expression level and the number of divisions cells undergone. Notice that this is an important aspect for a long-term follow up of the CFSE-labelled populations as the correspondence between the CFSE intensity range and the division generation can be heavily biased by the overall loss of the label over time and by the initial heterogeneity of the labelled cell population; (*iii*) it allows to estimate the kinetic parameters of cell proliferation and death as functions of the marker expression level (and hence of the number of cell divisions).

Modelling with hyperbolic PDEs, being used in the context of data-driven parameter identification, presents a significant computational challenge due to the hyperbolic nature of the equations and due to the large size of the discretized problem. To our knowledge, no publicly available software package exists which deals with optimization of hyperbolic PDE models. We estimate the distributed parameters of the proposed model following the maximum likelihood approach and using the direct search Nelder-Mead simplex method applied to a finite dimensional approximation of the original infinite dimensional optimization problem. The initial-boundary value problem is solved with a Matlab program by Shampine [22], which implements the well established second order Richtmyer's two-step variant of the Lax-Wendroff method. Because this program is fully vectorized, it allows very fast execution, which is otherwise difficult to achieve in Matlab. This is especially important when solving a PDE in an optimization loop. Using two original CFSE data sets, we demonstrate the biological consistency of the proposed label-structured model and compare its predictions with the predictions of the ODE (ordinary differential equation) compartmental model published recently [12].

The outline of this paper is as follows. In the next section we formulate the label-structured cell populations model. In section ”CFSE data” we describe two original sets of data on in vitro growth of human CFSE-labelled T-lymphocytes and the preprocessing of the corresponding CFSE histograms used in this study. The major aspects and the numerical treatment of the distributed parameter identification problem are presented in sections ”Parameter estimation” and ”Numerical procedure”. Results of the application of the proposed model to the analysis of the turnover parameters of proliferating cells from the CFSE intensity histograms for the two data sets are presented in section ”Applications to CFSE assay”. Here we also compare the performance of the proposed PDE model and the compartmental ODE model. Finally, we discuss the major advantages and the bottlenecks of the proposed approach.

## Label-structured cell populations model

In this section we introduce the mathematical model for the dynamics of lymphocyte populations in the CFSE proliferation assay. We consider a population of cells which are structured according to a single variable *x *that characterizes the CFSE expression level in terms of units of intensity, *UI*. Therefore the amount of CFSE label is treated as a continuous variable. The state of the population at time *t *is described by the distribution (density) function *n*(*t*, *x*)(*cell*/*UI*), so that the number of cells with the CFSE intensity between *x*_1 _and *x*_2 _is given by ∫x1x2n(t,x)dx
 MathType@MTEF@5@5@+=feaafiart1ev1aaatCvAUfKttLearuWrP9MDH5MBPbIqV92AaeXatLxBI9gBaebbnrfifHhDYfgasaacH8akY=wiFfYdH8Gipec8Eeeu0xXdbba9frFj0=OqFfea0dXdd9vqai=hGuQ8kuc9pgc9s8qqaq=dirpe0xb9q8qiLsFr0=vr0=vr0dc8meaabaqaciaacaGaaeqabaqabeGadaaakeaadaWdXaqaaiabd6gaUjabcIcaOiabdsha0jabcYcaSiabdIha4jabcMcaPaWcbaGaemiEaG3aaSbaaWqaaiabigdaXaqabaaaleaacqWG4baEdaWgaaadbaGaeGOmaidabeaaa0Gaey4kIipakiabdsgaKjabdIha4baa@3DC3@

At the beginning of the follow-up experiment, the lymphocyte population is stained with CFSE giving rise to the initial (starting) distribution of cells with respect to the CFSE fluorescence. The following phenomenological features of the label-structured lymphocyte proliferation have to be taken into account by the model for the dynamics of the distribution of labelled cells ([5-7,23]):

• During cell division CFSE is partitioned equally between daughter cells;

• The fluorescence intensity of labeled cells declines slowly over time due to catabolism [5,6,24];

• Each CFSE division peak represents a cohort of cells that entered their first division at approximately the same time;

• As the cells proliferate, the initially bell-shaped distribution of the CFSE fluorescence in the population becomes multimodal, moving over time to lower values of *x*. The histograms of the CFSE intensity provide profiles for cell divisions;

• As the dividing cell population approaches the autofluorescence level of unlabelled cells, the division peaks start to compress, thus limiting the number of divisions that can be followed. Usually cells are stained to an intensity of about 10^3 ^times brighter than their autofluorescence, so that up to 10 divisions can be permitted while maintaining both the parental and the final generation intensities all on scale.

The label-structured cell population behavior can be expressed using a modification of the model proposed originally by Bell & Anderson for size-dependent cell population growth when reproduction occurs by fission into two equal parts [19]. We assume that the physiological parameters of cells (division and death rates) strongly correlate with the label expression level.

Let the initial CFSE distribution of cells at time *t*_0 _be given by the density function

*n*(*t*_0_, *x*) =: *n*_*0*_(*x*), *x *∈ [*x*_min_, *x*_max_].

This can be either the cell distribution at the start of the experiment (*t*_0 _= 0) or at some later time (*t*_0 _> 0). The evolution of the cell distribution *n*(*t*, *x*) is modelled by the following cell population balance one-dimensional hyperbolic PDE,

∂n∂t(t,x)−v(x)∂n∂x(t,x)=−(α(x)+β(x))n(t,x)+2γα(γx)n(t,γx),xmin⁡≤x≤xmax⁡/γ,∂n∂t(t,x)−v(x)∂n∂x(t,x)=−(α(x)+β(x))n(t,x),xmax⁡/γ≤x≤xmax⁡.
 MathType@MTEF@5@5@+=feaafiart1ev1aaatCvAUfKttLearuWrP9MDH5MBPbIqV92AaeXatLxBI9gBaebbnrfifHhDYfgasaacH8akY=wiFfYdH8Gipec8Eeeu0xXdbba9frFj0=OqFfea0dXdd9vqai=hGuQ8kuc9pgc9s8qqaq=dirpe0xb9q8qiLsFr0=vr0=vr0dc8meaabaqaciaacaGaaeqabaqabeGadaaakeaafaqaaeGabaaabaqbaeqabeGaaaqaamaalaaabaGaeyOaIyRaemOBa4gabaGaeyOaIyRaemiDaqhaaiabcIcaOiabdsha0jabcYcaSiabdIha4jabcMcaPiabgkHiTiabdAha2jabcIcaOiabdIha4jabcMcaPmaalaaabaGaeyOaIyRaemOBa4gabaGaeyOaIyRaemiEaGhaaiabcIcaOiabdsha0jabcYcaSiabdIha4jabcMcaPiabg2da9iabgkHiTiabcIcaOGGaciab=f7aHjabcIcaOiabdIha4jabcMcaPiabgUcaRiab=j7aIjabcIcaOiabdIha4jabcMcaPiabcMcaPiabd6gaUjabcIcaOiabdsha0jabcYcaSiabdIha4jabcMcaPiabgUcaRiabikdaYiab=n7aNjab=f7aHjabcIcaOiab=n7aNjabdIha4jabcMcaPiabd6gaUjabcIcaOiabdsha0jabcYcaSiab=n7aNjabdIha4jabcMcaPiabcYcaSaqaaiabdIha4naaBaaaleaacyGGTbqBcqGGPbqAcqGGUbGBaeqaaOGaeyizImQaemiEaGNaeyizImQaemiEaG3aaSbaaSqaaiGbc2gaTjabcggaHjabcIha4bqabaGccqGGVaWlcqWFZoWzcqGGSaalaaaabaqbaeqabeGaaaqaamaalaaabaGaeyOaIyRaemOBa4gabaGaeyOaIyRaemiDaqhaaiabcIcaOiabdsha0jabcYcaSiabdIha4jabcMcaPiabgkHiTiabdAha2jabcIcaOiabdIha4jabcMcaPmaalaaabaGaeyOaIyRaemOBa4gabaGaeyOaIyRaemiEaGhaaiabcIcaOiabdsha0jabcYcaSiabdIha4jabcMcaPiabg2da9iabgkHiTiabcIcaOiab=f7aHjabcIcaOiabdIha4jabcMcaPiabgUcaRiab=j7aIjabcIcaOiabdIha4jabcMcaPiabcMcaPiabd6gaUjabcIcaOiabdsha0jabcYcaSiabdIha4jabcMcaPiabcYcaSaqaaiabdIha4naaBaaaleaacyGGTbqBcqGGHbqycqGG4baEaeqaaOGaei4la8Iae83SdCMaeyizImQaemiEaGNaeyizImQaemiEaG3aaSbaaSqaaiGbc2gaTjabcggaHjabcIha4bqabaGccqGGUaGlaaaaaaaa@CAB2@

The first equation consists of the following terms:

*v*(*x*)∂*n*(*t*, *x*)/∂*x*, the advection term, describes the natural decay of the CFSE fluorescence intensity of the labelled cells with the rate *v*(*x*), *UI*/*hour*;

-(*α*(*x*) + *β*(*x*))*n*(*t*, *x*) describes the local disappearance of cells with the CFSE intensity *x *due to the division associated CFSE dilution and the death with *α*(*x*) ≥ 0 and *β*(*x*) ≥ 0 being the proliferation and death rates, respectively, both having the same unit 1/*hour*;

2*γα*(*γx*)*n*(*t*, *γx*) represents the birth of two cells due to division of the mother cell with the label intensity *γx*. The first factor accounts for the doubling of numbers, and the second for the difference by a factor *γ *in the size of the CFSE intervals to which daughter and mother cells belong. Indeed, those cells which originate from division of cells with CFSE in the range (*γx*, *γ*(*x *+ *dx*)) enter into the range (*x*, *x *+ *dx*).

Under the assumption of equal partition of the label between the two daughter cells and no death during the division one expects that *γ *= 2. This would ensure conservation of CFSE label, similar to the conservation of volume-size [19,20]. However, we allow the label partitioning parameter *γ *to take values smaller than 2 so that *x *<*γx *≤ 2*x*, in order to check the consistency of the assumptions with experimental data.

The above consideration applies to cells with levels of CFSE below the maximal initial staining *x*_max _divided by *γ*. The population dynamics of the cells with *x*_max_/*γ *<*x *≤ *x*_max _is governed by the second equation of model (2) without the source term. The division, death and transition rates, *α*(*x*), *β*(*x*) and *v*(*x*), of the structured population are assumed to be functions of (i.e., correlate with) the CFSE intensity. The precise dependence on *x *is not known a priori and will be estimated from the flow cytometry data.

The initial data for model (2) are given by (1) specifying the distribution of cells at time *t*_0_. The lack of cells with CFSE intensity above the given maximal value *x*_max _for all *t *> *t*_0 _is taken into account by the boundary condition

*n*(*t*, *x*_max_) = 0, *t *> *t*_0_.

The basic model (2) is formulated using the linear scale for the structure variable *x*. As the histograms obtained by flow cytometry use the base 10 logarithm of the marker expression level, we reformulate model (2) to deal directly with the transformed structure variable *z *:= log_10_*x*,

∂n∂t(t,z)−ν(z)∂n∂z(t,z)=−(α(z)+β(z))n(t,z)+2γα(z+log⁡10γ)n(t,z+log⁡10γ), zmin⁡≤z≤zmax⁡−log⁡10γ,∂n∂t(t,z)−ν(z)∂n∂x(t,z)=−(α(z)+β(z))n(t,z), zmax⁡−log⁡10γ≤z≤zmax⁡,
 MathType@MTEF@5@5@+=feaafiart1ev1aaatCvAUfKttLearuWrP9MDH5MBPbIqV92AaeXatLxBI9gBaebbnrfifHhDYfgasaacH8akY=wiFfYdH8Gipec8Eeeu0xXdbba9frFj0=OqFfea0dXdd9vqai=hGuQ8kuc9pgc9s8qqaq=dirpe0xb9q8qiLsFr0=vr0=vr0dc8meaabaqaciaacaGaaeqabaqabeGadaaakeaafaqaaeGabaaabaWaaSaaaeaacqGHciITcqWGUbGBaeaacqGHciITcqWG0baDaaGaeiikaGIaemiDaqNaeiilaWIaemOEaONaeiykaKIaeyOeI0ccciGae8xVd4MaeiikaGIaemOEaONaeiykaKYaaSaaaeaacqGHciITcqWGUbGBaeaacqGHciITcqWG6bGEaaGaeiikaGIaemiDaqNaeiilaWIaemOEaONaeiykaKIaeyypa0JaeyOeI0IaeiikaGIae8xSdeMaeiikaGIaemOEaONaeiykaKIaey4kaSIae8NSdiMaeiikaGIaemOEaONaeiykaKIaeiykaKIaemOBa4MaeiikaGIaemiDaqNaeiilaWIaemOEaONaeiykaKIaey4kaSIaeGOmaiJae83SdCMae8xSdeMaeiikaGIaemOEaONaey4kaSIagiiBaWMaei4Ba8Maei4zaC2aaSbaaSqaaiabigdaXiabicdaWaqabaGccqWFZoWzcqGGPaqkcqWGUbGBcqGGOaakcqWG0baDcqGGSaalcqWG6bGEcqGHRaWkcyGGSbaBcqGGVbWBcqGGNbWzdaWgaaWcbaGaeGymaeJaeGimaadabeaakiab=n7aNjabcMcaPiabcYcaSiabbccaGiabdQha6naaBaaaleaacyGGTbqBcqGGPbqAcqGGUbGBaeqaaOGaeyizImQaemOEaONaeyizImQaemOEaO3aaSbaaSqaaiGbc2gaTjabcggaHjabcIha4bqabaGccqGHsislcyGGSbaBcqGGVbWBcqGGNbWzdaWgaaWcbaGaeGymaeJaeGimaadabeaakiab=n7aNjabcYcaSaqaamaalaaabaGaeyOaIyRaemOBa4gabaGaeyOaIyRaemiDaqhaaiabcIcaOiabdsha0jabcYcaSiabdQha6jabcMcaPiabgkHiTiab=17aUjabcIcaOiabdQha6jabcMcaPmaalaaabaGaeyOaIyRaemOBa4gabaGaeyOaIyRaemiEaGhaaiabcIcaOiabdsha0jabcYcaSiabdQha6jabcMcaPiabg2da9iabgkHiTiabcIcaOiab=f7aHjabcIcaOiabdQha6jabcMcaPiabgUcaRiab=j7aIjabcIcaOiabdQha6jabcMcaPiabcMcaPiabd6gaUjabcIcaOiabdsha0jabcYcaSiabdQha6jabcMcaPiabcYcaSiabbccaGiabdQha6naaBaaaleaacyGGTbqBcqGGHbqycqGG4baEaeqaaOGaeyOeI0IagiiBaWMaei4Ba8Maei4zaC2aaSbaaSqaaiabigdaXiabicdaWaqabaGccqWFZoWzcqGHKjYOcqWG6bGEcqGHKjYOcqWG6bGEdaWgaaWcbaGagiyBa0MaeiyyaeMaeiiEaGhabeaakiabcYcaSaaaaaa@E78C@

where *ν*(*z*) = *v*(10^*z*^)/log(10)10^*z*^. The structured population balance model (4) is used for the description of the evolution of CFSE histograms and to estimate the division, death and transfer rates of labelled cell populations from CFSE proliferation assays.

## CFSE data

### CFSE intensity histograms of proliferating cell population

To investigate the appropriateness of the label-structured cell population model (4) and the developed parameter estimation procedure, two original data sets characterizing the evolution of CFSE distribution of proliferating cell cultures were used. The data sets were obtained from *in vitro *proliferation assay with human peripheral blood mononuclear cells (PBMC) as follows. The cells were labelled with CFSE at day 0. To induce the proliferation of T cells, two different activation stimuli were used:

• the mitogen stimulator phytohemagglutinin (PHA), which activates the T lymphocytes unspecifically, i.e., independent of a signal transduced by the T cell receptor (data set 1, considers the total CD4 and CD8 T cells);

• the antibodies against CD3 and CD28 receptors on T cells which provide signals similar to those transduced by the T cell receptor (data set 2, considers the CD4 T cells).

At regular times after the onset of cell proliferation the cells were harvested, stained with antibodies to CD4 or CD8 and analyzed by flow cytometry for CFSE expression level on individual cells. The total cell number in the proliferation culture was also quantified. The combination of CFSE labelling and flow cytometry allows one to generate the time series of histograms of CFSE distribution [5].

**Figure 2:**
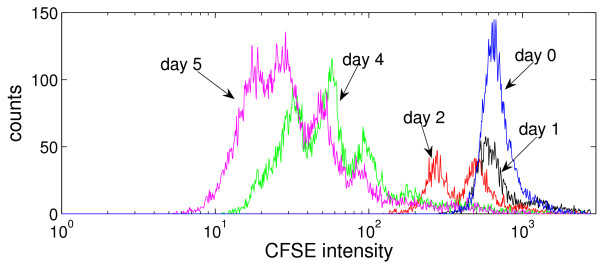
The original CFSE histograms at days 0,1,2,4,5 (data set 2).

Figure 2 shows the CFSE histograms for data set 2: the distribution of proliferating CFSE-labelled T cells according to the intensity of the CFSE label from the start of the experiment until day 5. Provided that the initial cell labelling is fairly homogeneous, each CFSE peak represents a cohort of cells that proceed synchronously through the division rounds. As cells proliferate the whole cell population moves, with respect to the CFSE fluorescence intensity, from right to left, demonstrating sequential loss of CFSE fluorescence with time. The observed fluctuating behavior of the measurements results from a superposition of a whole range of random processes, including cell counting, inherent heterogeneity of the cell shape in the population, background noise in the functioning of the physical elements constituting the FACS machine. To use such histograms of CFSE distributions in the numerical parameter estimation problem, a preprocessing of the data is required, cf. the next section.

In a standard approach, the CFSE fluorescence histograms are used to evaluate the fractions of T cells that have completed certain number of divisions [6,7]. This type of 'mean fluorescence intensity' data can be obtained either manually or by using various deconvolution techniques implemented in programs, such as ModFit (Verity Software), CellQuest (Becton Dickinson), CFSE Modeler (ScienceSpeak). The corresponding computer-based procedures require setting of the spacing between generations, i.e., marking the CFSE fluorescence intensities that separate consecutive generations of dividing cells. Note that when the starting population of cells exhibits a broad range of CFSE fluorescence, the division peaks can be not easily identifiable, making conventional division tracking analysis problematic [3,23,25]. The number of divisions which can be followed is limited by the autofluorescence of unlabelled cells. For the data we consider, the resolution of the division peaks is not possible after about 7 division cycles. We present and make use of the division number lumped CFSE distribution data, i.e., 'mean fluorescence intensity', in the last section for comparison of the parameter estimation results for the PDE and ODE based models of cell proliferation.

### Preprocessing of CFSE intensity histograms for parameter estimation

**Figure 3:**
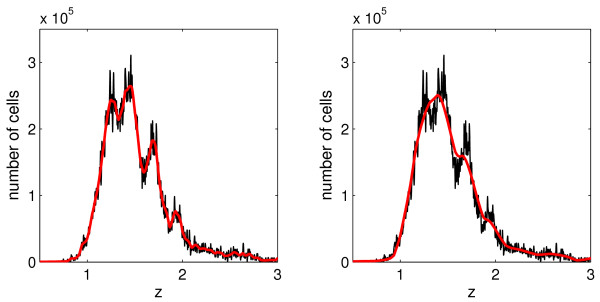
**The performance of the smoothing procedure for CFSE intensity histograms**. The original CFSE histogram (black curve) and two smoothed histograms (red curves) obtained by the algorithm in [26] using the smoothing factor (6) with *q *= 0.03 (left) and *q *= 0.05 (right).

Each of the histograms of CFSE-labelled cell counts obtained by flow cytometry at times *t*_*i*_, *i *= 0, 1, ..., *M*, can be considered as an array consisting of vectors Zi
 MathType@MTEF@5@5@+=feaafiart1ev1aaatCvAUfKttLearuWrP9MDH5MBPbIqV92AaeXatLxBI9gBaebbnrfifHhDYfgasaacH8akY=wiFfYdH8Gipec8Eeeu0xXdbba9frFj0=OqFfea0dXdd9vqai=hGuQ8kuc9pgc9s8qqaq=dirpe0xb9q8qiLsFr0=vr0=vr0dc8meaabaqaciaacaGaaeqabaqabeGadaaakeaatuuDJXwAK1uy0HwmaeHbfv3ySLgzG0uy0Hgip5wzaGabaiab=Lr8AnaaBaaaleaacqWGPbqAaeqaaaaa@39A5@, Ci∈ℝMi
 MathType@MTEF@5@5@+=feaafiart1ev1aaatCvAUfKttLearuWrP9MDH5MBPbIqV92AaeXatLxBI9gBaebbnrfifHhDYfgasaacH8akY=wiFfYdH8Gipec8Eeeu0xXdbba9frFj0=OqFfea0dXdd9vqai=hGuQ8kuc9pgc9s8qqaq=dirpe0xb9q8qiLsFr0=vr0=vr0dc8meaabaqaciaacaGaaeqabaqabeGadaaakeaat0uy0HwzTfgDPnwy1egaryqtHrhAL1wy0L2yHvdaiqaacqWFce=qdaWgaaWcbaGaemyAaKgabeaakiabgIGioprr1ngBPrwtHrhAYaqehuuDJXwAKbstHrhAGq1DVbacfaGae4xhHi1aaWbaaSqabeaacqWGnbqtdaWgaaadbaGaemyAaKgabeaaaaaaaa@48C9@ which correspond to the base 10 logarithm of the measured marker expression level, Zi:=[zi,1,...,zi,Mi]
 MathType@MTEF@5@5@+=feaafiart1ev1aaatCvAUfKttLearuWrP9MDH5MBPbIqV92AaeXatLxBI9gBaebbnrfifHhDYfgasaacH8akY=wiFfYdH8Gipec8Eeeu0xXdbba9frFj0=OqFfea0dXdd9vqai=hGuQ8kuc9pgc9s8qqaq=dirpe0xb9q8qiLsFr0=vr0=vr0dc8meaabaqaciaacaGaaeqabaqabeGadaaakeaatuuDJXwAK1uy0HwmaeHbfv3ySLgzG0uy0Hgip5wzaGabaiab=Lr8AnaaBaaaleaacqWGPbqAaeqaaOGaeiOoaOJaeyypa0Jaei4waSLaemOEaO3aaSbaaSqaaiabdMgaPjabcYcaSiabigdaXaqabaGccqGGSaalcqGGUaGlcqGGUaGlcqGGUaGlcqGGSaalcqWG6bGEdaWgaaWcbaGaemyAaKMaeiilaWIaemyta00aaSbaaWqaaiabdMgaPbqabaaaleqaaOGaeiyxa0faaa@4E1F@, and the numbers of counts Ci=[ci,1,...,ci,Mi]
 MathType@MTEF@5@5@+=feaafiart1ev1aaatCvAUfKttLearuWrP9MDH5MBPbIqV92AaeXatLxBI9gBaebbnrfifHhDYfgasaacH8akY=wiFfYdH8Gipec8Eeeu0xXdbba9frFj0=OqFfea0dXdd9vqai=hGuQ8kuc9pgc9s8qqaq=dirpe0xb9q8qiLsFr0=vr0=vr0dc8meaabaqaciaacaGaaeqabaqabeGadaaakeaat0uy0HwzTfgDPnwy1egaryqtHrhAL1wy0L2yHvdaiqaacqWFce=qdaWgaaWcbaGaemyAaKgabeaakiabg2da9iabcUfaBjabdogaJnaaBaaaleaacqWGPbqAcqGGSaalcqaIXaqmaeqaaOGaeiilaWIaeiOla4IaeiOla4IaeiOla4IaeiilaWIaem4yam2aaSbaaSqaaiabdMgaPjabcYcaSiabd2eannaaBaaameaacqWGPbqAaeqaaaWcbeaakiabc2faDbaa@4CCD@ associated with Zi
 MathType@MTEF@5@5@+=feaafiart1ev1aaatCvAUfKttLearuWrP9MDH5MBPbIqV92AaeXatLxBI9gBaebbnrfifHhDYfgasaacH8akY=wiFfYdH8Gipec8Eeeu0xXdbba9frFj0=OqFfea0dXdd9vqai=hGuQ8kuc9pgc9s8qqaq=dirpe0xb9q8qiLsFr0=vr0=vr0dc8meaabaqaciaacaGaaeqabaqabeGadaaakeaatuuDJXwAK1uy0HwmaeHbfv3ySLgzG0uy0Hgip5wzaGabaiab=Lr8AnaaBaaaleaacqWGPbqAaeqaaaaa@39A5@. Here *M*_*i *_stands for the number of mesh points at which the CFSE histogram at time *t*_*i *_is specified. To translate the flow cytometry counts data to cell numbers which are actually considered in model (4), we use the transformation

ni,j=ci,jNiFi,Fi=∫zmin⁡zmax⁡c˜i(z)dz,i=0,1,...,M,j=1,...,Mi,
 MathType@MTEF@5@5@+=feaafiart1ev1aaatCvAUfKttLearuWrP9MDH5MBPbIqV92AaeXatLxBI9gBaebbnrfifHhDYfgasaacH8akY=wiFfYdH8Gipec8Eeeu0xXdbba9frFj0=OqFfea0dXdd9vqai=hGuQ8kuc9pgc9s8qqaq=dirpe0xb9q8qiLsFr0=vr0=vr0dc8meaabaqaciaacaGaaeqabaqabeGadaaakeaafaqabeqaeaaaaeaacqWGUbGBdaWgaaWcbaGaemyAaKMaeiilaWIaemOAaOgabeaakiabg2da9maalaaabaGaem4yam2aaSbaaSqaaiabdMgaPjabcYcaSiabdQgaQbqabaGccqWGobGtdaWgaaWcbaGaemyAaKgabeaaaOqaaiabdAeagnaaBaaaleaacqWGPbqAaeqaaaaakiabcYcaSaqaaiabdAeagnaaBaaaleaacqWGPbqAaeqaaOGaeyypa0Zaa8qmaeaacuWGJbWygaacamaaBaaaleaacqWGPbqAaeqaaOGaeiikaGIaemOEaONaeiykaKIaemizaqMaemOEaOhaleaacqWG6bGEdaWgaaadbaGagiyBa0MaeiyAaKMaeiOBa4gabeaaaSqaaiabdQha6naaBaaameaacyGGTbqBcqGGHbqycqGG4baEaeqaaaqdcqGHRiI8aOGaeiilaWcabaGaemyAaKMaeyypa0JaeGimaaJaeiilaWIaeGymaeJaeiilaWIaeiOla4IaeiOla4IaeiOla4IaeiilaWIaemyta0KaeiilaWcabaGaemOAaOMaeyypa0JaeGymaeJaeiilaWIaeiOla4IaeiOla4IaeiOla4IaeiilaWIaemyta00aaSbaaSqaaiabdMgaPbqabaGccqGGSaalaaaaaa@7092@

where *N*_*i *_is the total number of cells at time *t*_*i *_(available from the experiment) and c˜i
 MathType@MTEF@5@5@+=feaafiart1ev1aaatCvAUfKttLearuWrP9MDH5MBPbIqV92AaeXatLxBI9gBaebbnrfifHhDYfgasaacH8akY=wiFfYdH8Gipec8Eeeu0xXdbba9frFj0=OqFfea0dXdd9vqai=hGuQ8kuc9pgc9s8qqaq=dirpe0xb9q8qiLsFr0=vr0=vr0dc8meaabaqaciaacaGaaeqabaqabeGadaaakeaacuWGJbWygaacamaaBaaaleaacqWGPbqAaeqaaaaa@2F91@ is a continuous approximation of the vector Ci
 MathType@MTEF@5@5@+=feaafiart1ev1aaatCvAUfKttLearuWrP9MDH5MBPbIqV92AaeXatLxBI9gBaebbnrfifHhDYfgasaacH8akY=wiFfYdH8Gipec8Eeeu0xXdbba9frFj0=OqFfea0dXdd9vqai=hGuQ8kuc9pgc9s8qqaq=dirpe0xb9q8qiLsFr0=vr0=vr0dc8meaabaqaciaacaGaaeqabaqabeGadaaakeaat0uy0HwzTfgDPnwy1egaryqtHrhAL1wy0L2yHvdaiqaacqWFce=qdaWgaaWcbaGaemyAaKgabeaaaaa@39AB@ defined on the mesh Zi
 MathType@MTEF@5@5@+=feaafiart1ev1aaatCvAUfKttLearuWrP9MDH5MBPbIqV92AaeXatLxBI9gBaebbnrfifHhDYfgasaacH8akY=wiFfYdH8Gipec8Eeeu0xXdbba9frFj0=OqFfea0dXdd9vqai=hGuQ8kuc9pgc9s8qqaq=dirpe0xb9q8qiLsFr0=vr0=vr0dc8meaabaqaciaacaGaaeqabaqabeGadaaakeaatuuDJXwAK1uy0HwmaeHbfv3ySLgzG0uy0Hgip5wzaGabaiab=Lr8AnaaBaaaleaacqWGPbqAaeqaaaaa@39A5@. *F*_*i *_is the total number of cell counts at time *t*_*i*_. Figure 3 shows an example of such transformed histogram, describing the labelled cell distribution that corresponds to the flow cytometry data set 2 for day 5.

A direct use of such fluctuating histogram data for numerical parameter estimation might lead to the following major difficulties: (*i*) the possibility of overfitting, when the measurement noise rather than the true dynamics is approximated; (*ii*) the emergence of discontinuities in the computed model solution due to a discontinuous initial cell distribution function, as suggested by the flow cytometry histogram. Overall, for the parameter estimation we need to infer the underlying cell distribution densities *n*(*t*_*i*_, *z*) from which the histograms of CFSE counts were sampled. The functional approximation allows one to make predictions about the CFSE-labelled cell density for the *z *coordinate where cells have not been observed. Because the density distribution is supposed to be a continuous function, the corresponding estimation problem involves some regularization procedure.

To find a continuous approximation for the histograms and to smooth the data, we used an algorithm proposed in [26], which is closely related to the Tikhonov regularization process [27]. In this approach a user-specified parameter *τ*, called the smoothing factor, controls the level of smoothing, such that the average squared deviation of the approximating function from the corresponding original position is limited to *τ*/*k*, with *k *being the number of mesh points in the histogram. To ensure a uniform level of smoothing for the whole series of histograms data available at times *t*_*i *_(which differ in the number of data points *M*_*i *_and the cell numbers *n*_*i*, *j*_) we used the following smoothing parameter *τ*_*i*_,

τi=miai2,ai=qmax⁡j(ni,j),i=1,...,Mi.
 MathType@MTEF@5@5@+=feaafiart1ev1aaatCvAUfKttLearuWrP9MDH5MBPbIqV92AaeXatLxBI9gBaebbnrfifHhDYfgasaacH8akY=wiFfYdH8Gipec8Eeeu0xXdbba9frFj0=OqFfea0dXdd9vqai=hGuQ8kuc9pgc9s8qqaq=dirpe0xb9q8qiLsFr0=vr0=vr0dc8meaabaqaciaacaGaaeqabaqabeGadaaakeaafaqabeqadaaabaacciGae8hXdq3aaSbaaSqaaiabdMgaPbqabaGccqGH9aqpcqWGTbqBdaWgaaWcbaGaemyAaKgabeaakiabdggaHnaaDaaaleaacqWGPbqAaeaacqaIYaGmaaGccqGGSaalaeaacqWGHbqydaWgaaWcbaGaemyAaKgabeaakiabg2da9iabdghaXnaaxababaGagiyBa0MaeiyyaeMaeiiEaGhaleaacqWGQbGAaeqaaOGaeiikaGIaemOBa42aaSbaaSqaaiabdMgaPjabcYcaSiabdQgaQbqabaGccqGGPaqkcqGGSaalaeaacqWGPbqAcqGH9aqpcqaIXaqmcqGGSaalcqGGUaGlcqGGUaGlcqGGUaGlcqGGSaalcqWGnbqtdaWgaaWcbaGaemyAaKgabeaaaaGccqGGUaGlaaa@56EE@

Here *q *defines the ”global” level of smoothing and *m*_*i *_stands for the number of measurements with *n*_*i*, *j *_> *a*_*i *_in the histogram being smoothed. The performance of the continuous smoothing procedure is presented in Fig. 3 for two choices of the parameter *q*. Note that a moderate level of smoothing (*q *= 0.03) preserves important features of the data (the division associated peaks), while *q *= 0.05 leads to oversmoothing (information loss) as manifested by the disappearance of the division cohort structure presented in the histogram. In our study we used *q *= 0.03.

The histograms obtained by flow cytometry cover the whole range of the CFSE fluorescence *x *from 1 to 10^4^. In particular, the starting population of undivided cells can spread up to the upper end of 10^4^units. We did not consider the tiny fraction of cells which differ substantially in their CFSE intensity from the bulk population of homogeneously stained cells. These CFSE bright cells might represent a measurement noise rather than genuine cells as they remain in the same area of the histogram at later observation times. Therefore, for parameter estimation we assumed that there is some maximum CFSE intensity *z*_max_, which depends on the initial staining of cells. This upper level of fluorescence was prescribed specifically for data sets 1 and 2.

## Parameter estimation

The population balance model (4), describing the distribution of cells *n*(*t*, *z*) structured according to the log_10_-transformed CFSE intensity, depends on the unknown rate functions of cell division *α*(*z*), death *β*(*z*) and the label loss *ν*(*z*). The identification of these functions from the observed CFSE histograms, using some measure of closeness of the model solution to the observations, represents an inverse problem. This problem is characterized by a finite set of observations *n*_*i*, *j*_and an infinite-dimensional space P
 MathType@MTEF@5@5@+=feaafiart1ev1aaatCvAUfKttLearuWrP9MDH5MBPbIqV92AaeXatLxBI9gBaebbnrfifHhDYfgasaacH8akY=wiFfYdH8Gipec8Eeeu0xXdbba9frFj0=OqFfea0dXdd9vqai=hGuQ8kuc9pgc9s8qqaq=dirpe0xb9q8qiLsFr0=vr0=vr0dc8meaabaqaciaacaGaaeqabaqabeGadaaakeaacqqGqbauaaa@2DD3@ of the functions to be estimated. Following a general approach to the numerical solution of the parameter estimation problem for distributed parameter systems [28-33], we need to parameterize the elements of the function space P
 MathType@MTEF@5@5@+=feaafiart1ev1aaatCvAUfKttLearuWrP9MDH5MBPbIqV92AaeXatLxBI9gBaebbnrfifHhDYfgasaacH8akY=wiFfYdH8Gipec8Eeeu0xXdbba9frFj0=OqFfea0dXdd9vqai=hGuQ8kuc9pgc9s8qqaq=dirpe0xb9q8qiLsFr0=vr0=vr0dc8meaabaqaciaacaGaaeqabaqabeGadaaakeaacqqGqbauaaa@2DD3@ in order to represent them by a finite set of parameters and to select the cost functional.

To avoid imposing a particular shape of the functions *α*(*z*) and *β*(*z*), we approximate these functions using piecewise monotone cubic interpolation through the points (*z*_*k*_, *a*_*k*_) and (*z*_*k*_, *b*_*k*_), respectively, with some *z*_*k *_∈ [*z*_min_, *z*_max_], *k *= 1, ..., *L*,

αL(z)=∑j=1Lajφj(z),βL(z)=∑j=1Lbjφj(z),z∈[zmin⁡,zmax⁡],
 MathType@MTEF@5@5@+=feaafiart1ev1aaatCvAUfKttLearuWrP9MDH5MBPbIqV92AaeXatLxBI9gBaebbnrfifHhDYfgasaacH8akY=wiFfYdH8Gipec8Eeeu0xXdbba9frFj0=OqFfea0dXdd9vqai=hGuQ8kuc9pgc9s8qqaq=dirpe0xb9q8qiLsFr0=vr0=vr0dc8meaabaqaciaacaGaaeqabaqabeGadaaakeaafaqabeqadaaabaacciGae8xSde2aaSbaaSqaaiabdYeambqabaGccqGGOaakcqWG6bGEcqGGPaqkcqGH9aqpdaaeWbqaaiabdggaHnaaBaaaleaacqWGQbGAaeqaaOGae8NXdy2aaSbaaSqaaiabdQgaQbqabaGccqGGOaakcqWG6bGEcqGGPaqkaSqaaiabdQgaQjabg2da9iabigdaXaqaaiabdYeambqdcqGHris5aOGaeiilaWcabaGae8NSdi2aaSbaaSqaaiabdYeambqabaGccqGGOaakcqWG6bGEcqGGPaqkcqGH9aqpdaaeWbqaaiabdkgaInaaBaaaleaacqWGQbGAaeqaaOGae8NXdy2aaSbaaSqaaiabdQgaQbqabaGccqGGOaakcqWG6bGEcqGGPaqkaSqaaiabdQgaQjabg2da9iabigdaXaqaaiabdYeambqdcqGHris5aOGaeiilaWcabaGaemOEaONaeyicI4Saei4waSLaemOEaO3aaSbaaSqaaiGbc2gaTjabcMgaPjabc6gaUbqabaGccqGGSaalcqWG6bGEdaWgaaWcbaGagiyBa0MaeiyyaeMaeiiEaGhabeaakiabc2faDjabcYcaSaaaaaa@6FEE@

Here *φ*_*j *_are cubic polynomials, such that *φ*_*j*_(*z*_*j*_) = 1, *φ*_*j*_(*z*_*k*_) = 0 for *j *≠ *k*, and hence *α*_*L*_(*z*_*k*_) = *a*_*k*_, *β*_*L*_(*z*_*k*_) = *b*_*k*_, *k *= 1, ..., *L*. Elements of the vectors a={ak}1L
 MathType@MTEF@5@5@+=feaafiart1ev1aaatCvAUfKttLearuWrP9MDH5MBPbIqV92AaeXatLxBI9gBaebbnrfifHhDYfgasaacH8akY=wiFfYdH8Gipec8Eeeu0xXdbba9frFj0=OqFfea0dXdd9vqai=hGuQ8kuc9pgc9s8qqaq=dirpe0xb9q8qiLsFr0=vr0=vr0dc8meaabaqaciaacaGaaeqabaqabeGadaaakeaaieqacqWFHbqycqGH9aqpcqGG7bWEcqWGHbqydaWgaaWcbaGaem4AaSgabeaakiabc2ha9naaDaaaleaacqaIXaqmaeaacqWGmbataaaaaa@3721@ and b={bk}1L
 MathType@MTEF@5@5@+=feaafiart1ev1aaatCvAUfKttLearuWrP9MDH5MBPbIqV92AaeXatLxBI9gBaebbnrfifHhDYfgasaacH8akY=wiFfYdH8Gipec8Eeeu0xXdbba9frFj0=OqFfea0dXdd9vqai=hGuQ8kuc9pgc9s8qqaq=dirpe0xb9q8qiLsFr0=vr0=vr0dc8meaabaqaciaacaGaaeqabaqabeGadaaakeaaieqacqWFIbGycqGH9aqpcqGG7bWEcqWGIbGydaWgaaWcbaGaem4AaSgabeaakiabc2ha9naaDaaaleaacqaIXaqmaeaacqWGmbataaaaaa@3725@ are the unknowns to be estimated.

For the rate function *ν*(*z*), we consider two plausible variants:

ν(z)≡candν(z)=clog⁡(10)10z,c∈ℝ+,z∈[zmin⁡,zmax⁡].
 MathType@MTEF@5@5@+=feaafiart1ev1aaatCvAUfKttLearuWrP9MDH5MBPbIqV92AaeXatLxBI9gBaebbnrfifHhDYfgasaacH8akY=wiFfYdH8Gipec8Eeeu0xXdbba9frFj0=OqFfea0dXdd9vqai=hGuQ8kuc9pgc9s8qqaq=dirpe0xb9q8qiLsFr0=vr0=vr0dc8meaabaqaciaacaGaaeqabaqabeGadaaakeaafaqabeqafaaaaeaaiiGacqWF9oGBcqGGOaakcqWG6bGEcqGGPaqkcqGHHjIUcqWGJbWyaeaacqqGHbqycqqGUbGBcqqGKbazaeaacqWF9oGBcqGGOaakcqWG6bGEcqGGPaqkcqGH9aqpdaWcaaqaaiabdogaJbqaaiGbcYgaSjabc+gaVjabcEgaNjabcIcaOiabigdaXiabicdaWiabcMcaPiabigdaXiabicdaWmaaCaaaleqabaGaemOEaOhaaaaakiabcYcaSaqaaiabdogaJjabgIGioprr1ngBPrwtHrhAYaqeguuDJXwAKbstHrhAGq1DVbaceaGae4xhHi1aaWbaaSqabeaacqGHRaWkaaGccqGGSaalaeaacqWG6bGEcqGHiiIZcqGGBbWwcqWG6bGEdaWgaaWcbaGagiyBa0MaeiyAaKMaeiOBa4gabeaakiabcYcaSiabdQha6naaBaaaleaacyGGTbqBcqGGHbqycqGG4baEaeqaaOGaeiyxa0LaeiOla4caaaaa@6E9C@

In terms of the CFSE fluorescence level *x*, cf. model (2), the first case assumes that the rate of label decay is directly proportional to the amount of label expressed on the cell: *v*(*x*) = *cx *log 10, while the second one implies that the CFSE loss does not depend on its level on the cells: *v*(*x*) ≡ *c*, *x *∈ [*x*_min_, *x*_max_].

Using the above parametrization, the original infinite dimensional problem of identifying the rate functions reduces to a finite dimensional one over a vector of parameters,

**p **:= [**a**, **b**, *c*, *γ*] ∈ ℝ^2*L*+2^.

The implementation details of the rate functions approximation are presented in the section ”Applications to CFSE assay” below.

To estimate the vector of best-fit parameters p*, we follow a maximum likelihood approach and seek for the parameter values which maximize the probability of observing the experimental data *n*_*i*, *j *_provided that the true values are specified by the model solution *n*(*t*, *z*; p*). The choice of the probability function should take into account the statistical nature of the observation errors. Because the statistical characterization of the CFSE fluorescence histograms for growing populations of cells is a poorly analyzed issue, we follow the principle stated in [34]: ”...in the absence of any other information the Central Limit Theorem tells us that the most reasonable choice for the distribution of a random variable is Gaussian.” Therefore, we assume that (*i*) the observational errors, i.e., the residuals defined as a difference between observed and model-predicted values, are normally distributed; (*ii*) the errors in observations at successive times are independent; (*iii*) the errors in cell counts for consecutive label bins are independent ((*ii*) – (*iii*) imply that the errors in the components of the state vector are independent); (*iv*) the variance of observation errors (*σ*^2^) is the same for all the state variables, observation times and label expression level.

Under the above assumptions the maximization of the log-likelihood function reduces

ln(ℒ
 MathType@MTEF@5@5@+=feaafiart1ev1aaatCvAUfKttLearuWrP9MDH5MBPbIqV92AaeXatLxBI9gBaebbnrfifHhDYfgasaacH8akY=wiFfYdH8Gipec8Eeeu0xXdbba9frFj0=OqFfea0dXdd9vqai=hGuQ8kuc9pgc9s8qqaq=dirpe0xb9q8qiLsFr0=vr0=vr0dc8meaabaqaciaacaGaaeqabaqabeGadaaakeaat0uy0HwzTfgDPnwy1egaryqtHrhAL1wy0L2yHvdaiqaacqWFsectaaa@376D@(**p**; *σ*)) = -0.5(*n*_*d *_ln(2*π*) + *n*_*d *_ln(*σ*^2^) + *σ*^-2^Φ(**p**))

to the minimization of the ordinary least-squares function, see for details [35],

Φ(p)=∑i=0M∑j=1Mi(ni,j−n(ti,zi,j;p))2,
 MathType@MTEF@5@5@+=feaafiart1ev1aaatCvAUfKttLearuWrP9MDH5MBPbIqV92AaeXatLxBI9gBaebbnrfifHhDYfgasaacH8akY=wiFfYdH8Gipec8Eeeu0xXdbba9frFj0=OqFfea0dXdd9vqai=hGuQ8kuc9pgc9s8qqaq=dirpe0xb9q8qiLsFr0=vr0=vr0dc8meaabaqaciaacaGaaeqabaqabeGadaaakeaacqqHMoGrcqGGOaakieqacqWFWbaCcqGGPaqkcqGH9aqpdaaeWbqaamaaqahabaGaeiikaGIaemOBa42aaSbaaSqaaiabdMgaPjabcYcaSiabdQgaQbqabaGccqGHsislcqWGUbGBcqGGOaakcqWG0baDdaWgaaWcbaGaemyAaKgabeaakiabcYcaSiabdQha6naaBaaaleaacqWGPbqAcqGGSaalcqWGQbGAaeqaaGqaaOGae43oaSJae8hCaaNaeiykaKIaeiykaKYaaWbaaSqabeaacqaIYaGmaaaabaGaemOAaOMaeyypa0JaeGymaedabaGaemyta00aaSbaaWqaaiabdMgaPbqabaaaniabggHiLdaaleaacqWGPbqAcqGH9aqpcqaIWaamaeaacqWGnbqta0GaeyyeIuoakiabcYcaSaaa@59BD@

provided that *σ*^2 ^is assigned the value σ∗2
 MathType@MTEF@5@5@+=feaafiart1ev1aaatCvAUfKttLearuWrP9MDH5MBPbIqV92AaeXatLxBI9gBaebbnrfifHhDYfgasaacH8akY=wiFfYdH8Gipec8Eeeu0xXdbba9frFj0=OqFfea0dXdd9vqai=hGuQ8kuc9pgc9s8qqaq=dirpe0xb9q8qiLsFr0=vr0=vr0dc8meaabaqaciaacaGaaeqabaqabeGadaaakeaaiiGacqWFdpWCdaahaaWcbeqaaiabgEHiQmaaCaaameqabaGaeGOmaidaaaaaaaa@30B2@ = Φ(p*)/*n*_*d*_, where p* is the vector which gives a minimum to Φ(**p**) and nd:=∑i=1MMi
 MathType@MTEF@5@5@+=feaafiart1ev1aaatCvAUfKttLearuWrP9MDH5MBPbIqV92AaeXatLxBI9gBaebbnrfifHhDYfgasaacH8akY=wiFfYdH8Gipec8Eeeu0xXdbba9frFj0=OqFfea0dXdd9vqai=hGuQ8kuc9pgc9s8qqaq=dirpe0xb9q8qiLsFr0=vr0=vr0dc8meaabaqaciaacaGaaeqabaqabeGadaaakeaacqWGUbGBdaWgaaWcbaGaemizaqgabeaakiabcQda6iabg2da9maaqadabaGaemyta00aaSbaaSqaaiabdMgaPbqabaaabaGaemyAaKMaeyypa0JaeGymaedabaGaemyta0eaniabggHiLdaaaa@3AAF@ is the total number of scalar measurements. Relevant details of the computational treatment of the parameter estimation problem for the PDE model (4) are presented in the next section.

## Numerical procedure

The parameter estimation problem for hyperbolic PDEs is non-trivial due to the hyperbolic nature of the equations (possible discontinuity of solutions) and due to the large size of the discretized problem. Moreover, model (4) is not a standard differential equation due to the solution term *n*(*t*, *z *+ log_10 _*γ*) with the transformed argument *z *+ log_10 _*γ *To our knowledge, no publicly available software package exists which deals with optimization (parameter estimation in particular) of models described by hyperbolic PDEs. For parabolic PDEs, which, after a suitable space discretization, can be treated as large systems of ODEs, available optimization tools (software, numerical methods) for large-scale problems can be used.

Solutions of a hyperbolic PDE can be discontinuous at the characteristic curve. Due to the solution term *n*(*t*, *z *+ log_10 _*γ*) in model (4), the discontinuity of solutions at a point (*t*, *z*_0_) on the characteristic curve propagates to the points (*t*, *z*_*j*_), *z*_*j *_= *z*_0 _- *j *log_10 _*γ*, *j *= 1, 2, .... A discretization of the initial-boundary value problem (4) should take into account the hyperbolicity of the equations and it should be robust and efficient since it is used in an optimization loop during model parameter identification. Moreover, available optimization tools for large-scale problems are based on some variants of Newton's method, which involves the computation of derivatives of the objective function with respect to the parameters to be estimated. These derivatives may not exist for discontinuous solutions. Note also that the optimization technique based on variants of Newton's method is efficient only if a good initial guess for the estimated parameters is available. For our problem, a derivative free minimization method which is robust with respect to the initial guess is preferable. Below we outline the numerical methods used and computational details of the problem under study.

### The initial-boundary value problem

To solve the initial-boundary value problem (IBVP) for model (4), we use the Matlab program hpde by L. Shampine developed for systems of first order hyperbolic PDEs in one space variable [22]. This program implements the well established second order Richtmyer's two-step variant of the Lax-Wendroff method (LxW) [36]. This method is dispersive and therefore the software contains the possibility to apply after each time step a nonlinear filter [37] to reduce the total variation of the numerical solution. When the solution is smooth, filtering has little effect, but the filter is helpful in dealing with the oscillations which are characteristic of the LxW scheme when the solution is discontinuous or has large gradients. The choice of this method was also influenced by its ability to be fully vectorized, which allows to speed up computations in Matlab significantly. This is especially important when solving a PDE in an optimization loop. To compute the solution term with the transformed argument *z *+ log_10 _*γ*, we modified the code hpde so that this term is interpolated, through its closest neighbors, preserving the second order accuracy of the LxW scheme.

To compute solutions of (4), we used a mesh *Z *:= [*z*_0_, *z*_1_, ..., *z*_*N*_] with equally spaced mesh points, Δ_*z *_:= *z*_*j *_- *z*_*j *- 1_, *j *= 1, ..., *N*. The initial data *n*_0_(*z*_*j*_) on the mesh *Z *are computed by interpolation of the given distribution of cells on the mesh Z0
 MathType@MTEF@5@5@+=feaafiart1ev1aaatCvAUfKttLearuWrP9MDH5MBPbIqV92AaeXatLxBI9gBaebbnrfifHhDYfgasaacH8akY=wiFfYdH8Gipec8Eeeu0xXdbba9frFj0=OqFfea0dXdd9vqai=hGuQ8kuc9pgc9s8qqaq=dirpe0xb9q8qiLsFr0=vr0=vr0dc8meaabaqaciaacaGaaeqabaqabeGadaaakeaatuuDJXwAK1uy0HwmaeHbfv3ySLgzG0uy0Hgip5wzaGabaiab=Lr8AnaaBaaaleaacqaIWaamaeqaaaaa@3938@ at time *t *= *t*_0_, using the Matlab code interp1 with a shape-preserving piecewise cubic interpolation. The Courant-Friedrichs-Lewy (CFL) condition

ΔtΔzmax⁡z∈Zν(z)<1
 MathType@MTEF@5@5@+=feaafiart1ev1aaatCvAUfKttLearuWrP9MDH5MBPbIqV92AaeXatLxBI9gBaebbnrfifHhDYfgasaacH8akY=wiFfYdH8Gipec8Eeeu0xXdbba9frFj0=OqFfea0dXdd9vqai=hGuQ8kuc9pgc9s8qqaq=dirpe0xb9q8qiLsFr0=vr0=vr0dc8meaabaqaciaacaGaaeqabaqabeGadaaakeaadaWcaaqaaiabfs5aenaaBaaaleaacqWG0baDaeqaaaGcbaGaeuiLdq0aaSbaaSqaaiabdQha6bqabaaaaOWaaCbeaeaacyGGTbqBcqGGHbqycqGG4baEaSqaaiabdQha6jabgIGiolabdQfaAbqabaacciGccqWF9oGBcqGGOaakcqWG6bGEcqGGPaqkcqGH8aapcqaIXaqmaaa@426B@

is a sufficient stability condition for the LxW scheme. To determine the time step in the PDE discretization, we use the CFL condition with safety factor 0.9,

Δt=0.9Δz/max⁡z∈Zν(z).
 MathType@MTEF@5@5@+=feaafiart1ev1aaatCvAUfKttLearuWrP9MDH5MBPbIqV92AaeXatLxBI9gBaebbnrfifHhDYfgasaacH8akY=wiFfYdH8Gipec8Eeeu0xXdbba9frFj0=OqFfea0dXdd9vqai=hGuQ8kuc9pgc9s8qqaq=dirpe0xb9q8qiLsFr0=vr0=vr0dc8meaabaqaciaacaGaaeqabaqabeGadaaakeaacqqHuoardaWgaaWcbaGaemiDaqhabeaakiabg2da9iabicdaWiabc6caUiabiMda5iabfs5aenaaBaaaleaacqWG6bGEaeqaaOGaei4la8YaaCbeaeaacyGGTbqBcqGGHbqycqGG4baEaSqaaiabdQha6jabgIGiolabdQfaAbqabaacciGccqWF9oGBcqGGOaakcqWG6bGEcqGGPaqkcqGGUaGlaaa@4609@

The time step is recomputed at each iteration of the optimization procedure since it depends on the estimated function *ν*(*z*).

It is well known that solutions of a hyperbolic PDE are discontinuous if the compatibility condition for the initial and boundary conditions is not fulfilled. In our case the compatibility condition reads as

*n*(0, *z*_max_) = *n*_0_(*z*_max_) = 0.

If *n*_0_(*z*) is the distribution of cells at the start of the experiment, i.e., *t*_0 _= 0, this condition is not fulfilled. In this case, the solution *n*(*t*, *z*) is discontinuous along the characteristic *z*(*t*) = *g*(*t*, *ν*(*z*)), defined by the ODE

dzdt=ν(z),z(0)=zmax⁡.
 MathType@MTEF@5@5@+=feaafiart1ev1aaatCvAUfKttLearuWrP9MDH5MBPbIqV92AaeXatLxBI9gBaebbnrfifHhDYfgasaacH8akY=wiFfYdH8Gipec8Eeeu0xXdbba9frFj0=OqFfea0dXdd9vqai=hGuQ8kuc9pgc9s8qqaq=dirpe0xb9q8qiLsFr0=vr0=vr0dc8meaabaqaciaacaGaaeqabaqabeGadaaakeaafaqabeqacaaabaWaaSaaaeaacqWGKbazcqWG6bGEaeaacqWGKbazcqWG0baDaaGaeyypa0dcciGae8xVd4MaeiikaGIaemOEaONaeiykaKIaeiilaWcabaGaemOEaONaeiikaGIaeGimaaJaeiykaKIaeyypa0JaemOEaO3aaSbaaSqaaiGbc2gaTjabcggaHjabcIha4bqabaGccqGGUaGlaaaaaa@450D@

**Figure 4:**
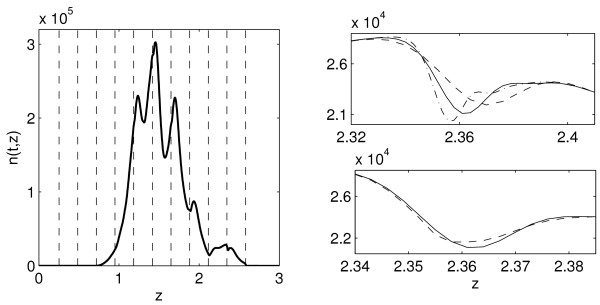
**Propagation of the discontinuities of the solution to model (4) and the effect of the mesh refinement and the filtering procedure**. Left: Solution *n*(*t*, *z*) of model (4) for *t *= 120 (hours) with the best-fit parameters estimated for data set 2. Dashed lines indicate positions of the discontinuities of the exact solution: zj∗
 MathType@MTEF@5@5@+=feaafiart1ev1aaatCvAUfKttLearuWrP9MDH5MBPbIqV92AaeXatLxBI9gBaebbnrfifHhDYfgasaacH8akY=wiFfYdH8Gipec8Eeeu0xXdbba9frFj0=OqFfea0dXdd9vqai=hGuQ8kuc9pgc9s8qqaq=dirpe0xb9q8qiLsFr0=vr0=vr0dc8meaabaqaciaacaGaaeqabaqabeGadaaakeaacqWG6bGEdaqhaaWcbaGaemOAaOgabaGaey4fIOcaaaaa@30A2@ = z0∗
 MathType@MTEF@5@5@+=feaafiart1ev1aaatCvAUfKttLearuWrP9MDH5MBPbIqV92AaeXatLxBI9gBaebbnrfifHhDYfgasaacH8akY=wiFfYdH8Gipec8Eeeu0xXdbba9frFj0=OqFfea0dXdd9vqai=hGuQ8kuc9pgc9s8qqaq=dirpe0xb9q8qiLsFr0=vr0=vr0dc8meaabaqaciaacaGaaeqabaqabeGadaaakeaacqWG6bGEdaqhaaWcbaGaeGimaadabaGaey4fIOcaaaaa@3033@ - *j *log_10 _*γ*, *j *= 0, 1, ..., 10, z0∗
 MathType@MTEF@5@5@+=feaafiart1ev1aaatCvAUfKttLearuWrP9MDH5MBPbIqV92AaeXatLxBI9gBaebbnrfifHhDYfgasaacH8akY=wiFfYdH8Gipec8Eeeu0xXdbba9frFj0=OqFfea0dXdd9vqai=hGuQ8kuc9pgc9s8qqaq=dirpe0xb9q8qiLsFr0=vr0=vr0dc8meaabaqaciaacaGaaeqabaqabeGadaaakeaacqWG6bGEdaqhaaWcbaGaeGimaadabaGaey4fIOcaaaaa@3033@ ≈ 2.58, *γ *≈ 1.71. Right (top): The effect of the mesh refinement on the computed solution in a neighborhood of the discontinuity at *z *≈ 2.347. Dashed, solid and dot-dashed curves indicate the solution computed using the mesh size *N *= 500, 1000, 2000, respectively. Right (bottom): The effect of the filtering procedure: the solution computed with and without the filtering (dashed, respectively solid curves). *N *= 1000.

If *ν*(*z*) is constant, this characteristic is *z *= *z*_max _- *νt*. Due to the solution term *n*(*t*, *z *+ log_10 _*γ*) in model (4), the discontinuity of the solution *n*(*t*, *z*) at z0∗
 MathType@MTEF@5@5@+=feaafiart1ev1aaatCvAUfKttLearuWrP9MDH5MBPbIqV92AaeXatLxBI9gBaebbnrfifHhDYfgasaacH8akY=wiFfYdH8Gipec8Eeeu0xXdbba9frFj0=OqFfea0dXdd9vqai=hGuQ8kuc9pgc9s8qqaq=dirpe0xb9q8qiLsFr0=vr0=vr0dc8meaabaqaciaacaGaaeqabaqabeGadaaakeaacqWG6bGEdaqhaaWcbaGaeGimaadabaGaey4fIOcaaaaa@3033@(*t*) = *g*(*t*, *ν*(z0∗
 MathType@MTEF@5@5@+=feaafiart1ev1aaatCvAUfKttLearuWrP9MDH5MBPbIqV92AaeXatLxBI9gBaebbnrfifHhDYfgasaacH8akY=wiFfYdH8Gipec8Eeeu0xXdbba9frFj0=OqFfea0dXdd9vqai=hGuQ8kuc9pgc9s8qqaq=dirpe0xb9q8qiLsFr0=vr0=vr0dc8meaabaqaciaacaGaaeqabaqabeGadaaakeaacqWG6bGEdaqhaaWcbaGaeGimaadabaGaey4fIOcaaaaa@3033@)) propagates to the points (*t*, zj∗
 MathType@MTEF@5@5@+=feaafiart1ev1aaatCvAUfKttLearuWrP9MDH5MBPbIqV92AaeXatLxBI9gBaebbnrfifHhDYfgasaacH8akY=wiFfYdH8Gipec8Eeeu0xXdbba9frFj0=OqFfea0dXdd9vqai=hGuQ8kuc9pgc9s8qqaq=dirpe0xb9q8qiLsFr0=vr0=vr0dc8meaabaqaciaacaGaaeqabaqabeGadaaakeaacqWG6bGEdaqhaaWcbaGaemOAaOgabaGaey4fIOcaaaaa@30A2@), with zj∗
 MathType@MTEF@5@5@+=feaafiart1ev1aaatCvAUfKttLearuWrP9MDH5MBPbIqV92AaeXatLxBI9gBaebbnrfifHhDYfgasaacH8akY=wiFfYdH8Gipec8Eeeu0xXdbba9frFj0=OqFfea0dXdd9vqai=hGuQ8kuc9pgc9s8qqaq=dirpe0xb9q8qiLsFr0=vr0=vr0dc8meaabaqaciaacaGaaeqabaqabeGadaaakeaacqWG6bGEdaqhaaWcbaGaemOAaOgabaGaey4fIOcaaaaa@30A2@ = z0∗
 MathType@MTEF@5@5@+=feaafiart1ev1aaatCvAUfKttLearuWrP9MDH5MBPbIqV92AaeXatLxBI9gBaebbnrfifHhDYfgasaacH8akY=wiFfYdH8Gipec8Eeeu0xXdbba9frFj0=OqFfea0dXdd9vqai=hGuQ8kuc9pgc9s8qqaq=dirpe0xb9q8qiLsFr0=vr0=vr0dc8meaabaqaciaacaGaaeqabaqabeGadaaakeaacqWG6bGEdaqhaaWcbaGaeGimaadabaGaey4fIOcaaaaa@3033@ - *j *log_10 _*γ*, *j *= 1, 2, ..., ∀*t*. This is illustrated in Fig. 4 (left).

Our experience with the solution of the IBVP for model (4), using the code hpde, has shown that oscillations in the computed solution, occurring due to the discontinuity of the exact solution, do not propagate significantly with respect to *z*. Hence, the accuracy of the computed solution is only influenced locally, see Fig. 4. With the mesh refinement, the amplitude of the oscillations grows, while the interval of the propagation of the oscillations decreases, cf. Fig. 4 (right, top). The filtering procedure of the hpde smoothes the oscillations, see Fig. 4 (right, bottom).

If the exact solution of model (4) is smooth, the order of accuracy of the computed solution on the interval [*z*_min_, *z*_max_] is uniform and corresponds to the order of the LxW scheme. This is the case for data set 1, for which the initial function is compatible with the boundary condition, *n*_0_(*z*_max_) = 0 for *t*_0 _= 72 hours. For *N *= 1000 the accuracy of the best-fit solution is about 10^-3 ^- 10^-2 ^and slowly decreases with time. For data set 2 the compatibility condition (13) is not fulfilled as *n*_0_(*z*_max_) ≠ 0 for *t*_0 _= 0. In this case the solution is discontinuous at points zj∗
 MathType@MTEF@5@5@+=feaafiart1ev1aaatCvAUfKttLearuWrP9MDH5MBPbIqV92AaeXatLxBI9gBaebbnrfifHhDYfgasaacH8akY=wiFfYdH8Gipec8Eeeu0xXdbba9frFj0=OqFfea0dXdd9vqai=hGuQ8kuc9pgc9s8qqaq=dirpe0xb9q8qiLsFr0=vr0=vr0dc8meaabaqaciaacaGaaeqabaqabeGadaaakeaacqWG6bGEdaqhaaWcbaGaemOAaOgabaGaey4fIOcaaaaa@30A2@ = z0∗
 MathType@MTEF@5@5@+=feaafiart1ev1aaatCvAUfKttLearuWrP9MDH5MBPbIqV92AaeXatLxBI9gBaebbnrfifHhDYfgasaacH8akY=wiFfYdH8Gipec8Eeeu0xXdbba9frFj0=OqFfea0dXdd9vqai=hGuQ8kuc9pgc9s8qqaq=dirpe0xb9q8qiLsFr0=vr0=vr0dc8meaabaqaciaacaGaaeqabaqabeGadaaakeaacqWG6bGEdaqhaaWcbaGaeGimaadabaGaey4fIOcaaaaa@3033@ - *j *log_10 _*γ*, *j *= 0, 1, ..., 10, see Fig. 4, and the above level of accuracy can only be achieved outside some small intervals around the discontinuity points.

Since model (4) is linear with respect to *n*(*t*, *z*), we scaled it by the factor 10^-5 ^to avoid the possible accuracy loss when dealing simultaneously with very large and small numbers in computations. To speed up the computations, the parameter estimation problem was treated in two stages. First we used a coarser mesh *Z *with *N *= 500 to solve the IBVP. Then the obtained best-fit parameter values were taken as a starting point to minimize the objective function using a finer mesh with *N *= 1000 to solve the IBVP.

### Parameterization of the estimated functions

According to the proposed parameterization (7) of the functions *α*(*z*) and *β*(*z*), the parameters to be estimated are elements of the vectors a={ak}1L
 MathType@MTEF@5@5@+=feaafiart1ev1aaatCvAUfKttLearuWrP9MDH5MBPbIqV92AaeXatLxBI9gBaebbnrfifHhDYfgasaacH8akY=wiFfYdH8Gipec8Eeeu0xXdbba9frFj0=OqFfea0dXdd9vqai=hGuQ8kuc9pgc9s8qqaq=dirpe0xb9q8qiLsFr0=vr0=vr0dc8meaabaqaciaacaGaaeqabaqabeGadaaakeaaieqacqWFHbqycqGH9aqpcqGG7bWEcqWGHbqydaWgaaWcbaGaem4AaSgabeaakiabc2ha9naaDaaaleaacqaIXaqmaeaacqWGmbataaaaaa@3721@ and b={bk}1L
 MathType@MTEF@5@5@+=feaafiart1ev1aaatCvAUfKttLearuWrP9MDH5MBPbIqV92AaeXatLxBI9gBaebbnrfifHhDYfgasaacH8akY=wiFfYdH8Gipec8Eeeu0xXdbba9frFj0=OqFfea0dXdd9vqai=hGuQ8kuc9pgc9s8qqaq=dirpe0xb9q8qiLsFr0=vr0=vr0dc8meaabaqaciaacaGaaeqabaqabeGadaaakeaaieqacqWFIbGycqGH9aqpcqGG7bWEcqWGIbGydaWgaaWcbaGaem4AaSgabeaakiabc2ha9naaDaaaleaacqaIXaqmaeaacqWGmbataaaaaa@3725@. Each pair (*a*_*k*_, *b*_*k*_) approximate the corresponding rate function at some value *z*_*k *_∈ [*z*_min_, *z*_max_] so that *α*_*L*_(*z*_*k*_) = *a*_*k *_and *β*_*L*_(*z*_*k*_) = *b*_*k*_, *k *= 1, ..., *L*. Values *z*_*k *_should be chosen such that all the consecutive divisions of cells could be captured properly. Hence the minimal value of *L *has to be larger than the maximal number of divisions cells have undergone. On the other hand, *L *should not be very large to treat the minimization problem efficiently. Values of *α*_*L*_(*z*) and *β*_*L*_(*z*) for *z *≠ *z*_*k *_were evaluated with the code interp1 by ashape-preserving piecewise cubic interpolation. In the following we omit the subscript *L *for simplicity.

For the initial parameterization we used *L *= 8. After the best-fit solution was found, the parameterization of *α*(*z*) and *β*(*z*) was updated as follows. For *α*(*z*), we added new points, thus introducing additional parameters to be estimated. The increase of *L *was restricted by the requirement that adding new parameters should allow one a better fit of the data, i.e., lead to a significant improvement in the computed minimum of the objective function. For data set 1, all estimated *b*_*k *_were close to some constant value. Therefore, we assumed that *β*(*z*) can be treated as a constant function. This simplifying assumption leads to a minor change in the values of the objective function (1%). For data set 2, all *b*_*k *_corresponding to *z*_*k *_< 2.5 were zeros and we fixed them to be zero.

### Minimization procedure

To solve the minimization problem, we use the Matlab code fminsearch implementing the Nelder-Mead simplex method. This method is a classical direct search algorithm that is widely used in case when the gradient of the objective function with respect to the estimated parameters cannot be evaluated. In our case the gradient, if it exists (i.e., if the solution of model (4) is continuous), can be computed numerically, but the computational cost is too large for the parameter estimation problem. As this method can trap in local minima for nonconvex objective functions, a number of runs with different initial guesses are necessary.

## Applications to CFSE assay

In this section we investigate the appropriateness of the proposed label-structured PDE model (4), using the two original data sets introduced in section ”CFSE data”. The performance of this model with respect to the data sets is further compared with that of the compartmental ODE model developed recently in [12].

### Mitogen-induced T cell proliferation

**Figure 5:**
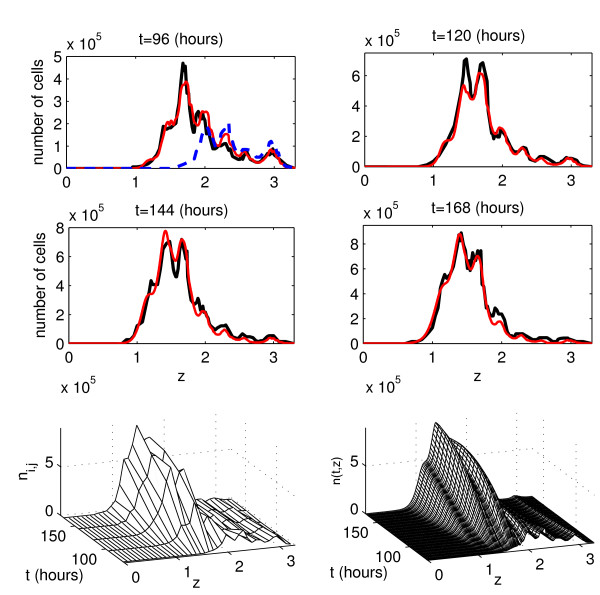
**The experimental data set 1 and the model solution corresponding to the best-fit parameter estimates**. Two first rows: Experimental data (black curves) and the best-fit solution of model (4) (red curves). The initial function is shown by a blue dashed curve. The last row presents the cell population surface: experimental data (left) and the model solution (right) as functions of time and the log_10_-transform of the marker expression level.

Figure 5 shows the experimental data set 1 and the solution of model (4) corresponding to the best-fit parameter estimates. The best-fit value of the objective function at the computed minimum is Φ ≈ 5.78 × 10^11^. The initial CFSE distribution is available at 72 hours after the beginning of the mitogen-induced T lymphocyte stimulation. One can see that both the CFSE label distributions, available at 96, 120, 144 and 168 hours, and the overall pattern of cell population surface are consistently reproduced by the model.

**Figure 6:**
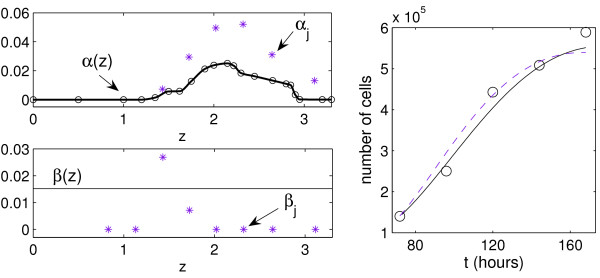
**For data set 1: the estimated rate functions and parameters of PDE model (4) and ODE model (15) and the kinetics of the total number of live lymphocytes predicted by both models**. Left: Dependence of the estimated turnover functions *α*(*z*) and *β*(*z*^) ^on the log_10_-transformed marker intensity. The best-fit estimates *a*_*k*_, *k *= 1, ..., 21, are indicated by circles. Stars specify the best-fit estimates for the birth and death parameters *α*_*j*_, *β*_*j*_, *j *= 0, ..., 5, of the ODE model (15). They are placed in the middle of the CFSE intervals which correspond to subsequent division numbers starting from 0. Right: The kinetics of the total number of live lymphocytes for data set 1 (circle) predicted by the PDE and ODE models (solid and dashed curves, respectively).

The best-fit estimates for the rate functions *α*(*z*) and *β*(*z*) are presented in Fig. 6 (left). The birth rate function *α*(*z*) appears to be bell-shaped. This is in agreement with our earlier results in [12], which showed a bell-shaped dependence of the birth rate of T lymphocytes on the number of divisions cells undergone. Following the proposed parameterization of the rate functions, the estimates of *b*_*k*_, *k *= 1, ..., *L*, appeared to be close to each other and Φ did not change much when they all were taken equal to the corresponding average value, overall suggesting that *β*(*z*) is a constant function of *z*. For the label decay rate *ν*(*z*), the second variant of parameterization in (8) with the best-fit estimate of the advection rate *c *≈ 0.11 provides a better approximation of the data by the model. Indeed, the respective values of the least squares function are 7.34·10^11 ^and 5.78·10^11^. The Akaike Information Criterion is also smaller for the second form of the advection rate (8678 versus 8603). This comparison implies that the label decay rate *ν*(*x*) as a function of the CFSE intensity per cell, cf. model (2), is predicted to be independent of *x*. The best fit estimate for the dilution parameter *γ *is *γ *≈ 1.93. In addition, the total population data observed experimentally and predicted by the model (the integral of the distribution density *n*(*t*, *z*) over the observed label intensity range) are shown in Fig. 6 (right). We observe that the label-structured model accurately reproduces the kinetics of mitogen-induced proliferation of T lymphocytes.

### CD3/CD28 antibody induced T cell proliferation

**Figure 7:**
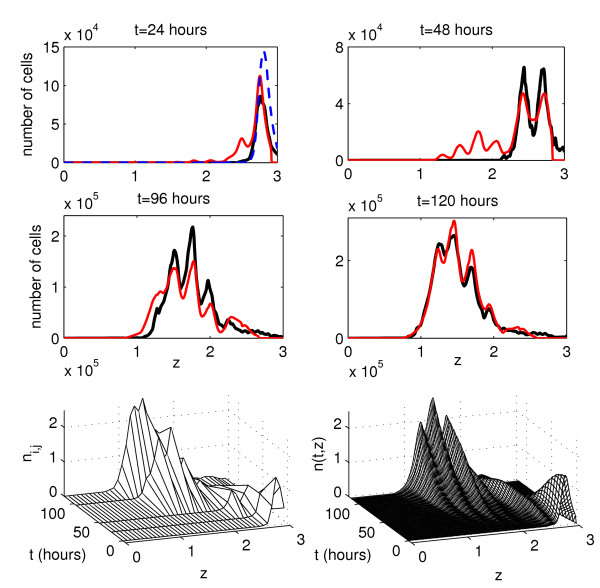
**The experimental data set 2 and the model solution corresponding to the best-fit parameter estimates**. Two first rows: Experimental data (black curves) and the best-fit solution of model (4) (red curves). The initial function is shown by a blue dashed curve. The last row presents the cell population surface: experimental data (left) and the model solution (right) as functions of time and the log_10_-transform of the marker expression level.

Figure 7 shows the experimental data set 2 on the stimulation of labelled T lymphocytes with antibodies against CD3 and CD28 cell surface receptors and the solution of model (4) corresponding to the best-fit parameter estimates. The best-fit value of the objective function at the computed minimum is Φ ≈ 1.14 × 10^12^. The initial CFSE distribution used corresponds to the beginning of the experiment. Overall, the kinetics of cell distribution are consistently reproduced by the model. The predicted shift in the cell distribution towards *z*-levels below 2 at 48 hours after the start of the experiment can be explained by the cell loss due to the culture handling, as described in the next paragraph.

**Figure 8:**
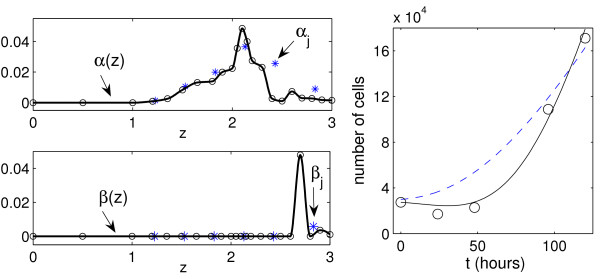
**For data set 2: the estimated rate functions and parameters of PDE model (4) and ODE model (15) and the kinetics of the total number of live lymphocytes predicted by both models**. Left: Dependence of the estimated cell turnover functions *α*(*z*) and *β*(*z*) on the log_10_-transformed marker intensity. The best-fit estimates *a*_*k*_, *b*_*k*_, *k *= 1, ..., 22, are indicated by circles. Stars specify the best-fit estimates for the birth and death parameters *α*_*j*_, *β*_*j*_, *j *= 0, ..., 5, of the ODE model (15). They are placed in the middle of the CFSE intervals which correspond to subsequent division numbers starting from 0. Right: The kinetics of the total number of live lymphocytes for data set 2 (circle) predicted by the PDE and ODE models (solid and dashed curves, respectively).

The best-fit estimates for the division and death rate functions *α*(*z*) and *β*(*z*) are presented in Fig. 8 (left). The function *α*(*z*) is bell-shaped but less monotone than in the case of data set 1. A sharp peak of the best-fit death rate *β*(*z*) around *z *≈ 2.6 (or CFSE ≈ 400) implies a large loss of cells during the first days of proliferation assay. Indeed, to perform the flow cytometry, the stimulating beads covered with antibodies need to be removed from the cell culture. During this separation stage, some of the cells which stay attached to the beads get also removed. This cell handling results in the predicted peak of the cell death rate and the spurious left tail of the cell distribution at 48 hours. Once the T cells are activated they detach from the beads to perform a series of programmed proliferation rounds and, therefore, one might expect that the effect of bead removal on the cell counts will reduce with time. For this data set, a constant advection rate *ν*(*z*) ≡ *c *with the best-fit estimate *c *≈ 3.5 × 10^-3 ^was enough to ensure a good approximation of the data by the model. This implies that the label loss rate *v*(*x*) in the original CFSE model (2) decreases with *x *as *v*(*x*) = *cx *log 10. The best fit estimate for label dilution parameter was again smaller than 2: *γ *≈ 1.71. Figure 8 (right) demonstrates that the model solution is consistent with the data on the growth of the total T cell population stimulated with antibodies to CD3/CD28.

### Comparison to compartmental ODE model

The label-structured PDE model allows to describe quantitatively the evolution of the heterogeneity of the proliferating T lymphocyte population with respect to the CFSE fluorescence intensity. It is instructive to compare the performance of this model with a mathematically simpler compartmental ODE model [12] for the proliferation of T cells heterogenous with respect to the division number. To this end, we evaluate how consistent this ODE model is with the data sets 1 and 2 reduced to the mean fluorescence intensities per generation. Using a uniform spacing between the consecutive cell generations, the CFSE histogram data suggest the division number cell distributions summarized in Table 1.

**Table 1:** The total number of live lymphocytes, *N*_*i*_, and the distribution of lymphocytes with respect to the number of divisions they have undergone, Nji
 MathType@MTEF@5@5@+=feaafiart1ev1aaatCvAUfKttLearuWrP9MDH5MBPbIqV92AaeXatLxBI9gBaebbnrfifHhDYfgasaacH8akY=wiFfYdH8Gipec8Eeeu0xXdbba9frFj0=OqFfea0dXdd9vqai=hGuQ8kuc9pgc9s8qqaq=dirpe0xb9q8qiLsFr0=vr0=vr0dc8meaabaqaciaacaGaaeqabaqabeGadaaakeaacqWGobGtdaqhaaWcbaGaemOAaOgabaGaemyAaKgaaaaa@30B6@, at the indicated times *t*_*i*_

Time days *t*_*i*_	Total number of live cells *N*_*i*_	Numbers of cells w.r.t. the number of divisions (*j*) they undergone Nji MathType@MTEF@5@5@+=feaafiart1ev1aaatCvAUfKttLearuWrP9MDH5MBPbIqV92AaeXatLxBI9gBaebbnrfifHhDYfgasaacH8akY=wiFfYdH8Gipec8Eeeu0xXdbba9frFj0=OqFfea0dXdd9vqai=hGuQ8kuc9pgc9s8qqaq=dirpe0xb9q8qiLsFr0=vr0=vr0dc8meaabaqaciaacaGaaeqabaqabeGadaaakeaacqWGobGtdaqhaaWcbaGaemOAaOgabaGaemyAaKgaaaaa@30B6@							
		
		0	1	2	3	4	5	6	7
data set 1									

3	1.4 × 10^5^	29358	22876	43372	39970	5208	98	14	
4	2.5 × 10^5^	16050	12600	22650	57025	96350	46950	2500	25
5	4.4 × 10^5^	14476	14784	25344	58652	141460	156290	32076	440
6	5.0 × 10^5^	13500	12150	24150	55000	137850	188950	69450	2150
7	5.7 × 10^5^	13509	12198	21603	51927	140560	232160	96102	3420

data set 2									

0	30000	30000							
1	20805	20623	182						
2	23725	13378	10042	305					
4	109218	4140	5504	16000	39276	36445	7845	8	
5	168156	3301	4012	9354	31713	60753	53486	5537	

The compartmental model considers the proliferation dynamics of cell populations. It assumes that the per capita proliferation and death rates of T lymphocytes, *α*_*j *_and *β*_*j*_, depend on the number of divisions the lymphocytes performed. The rate of change of the population of live lymphocytes having undergone *j *divisions (which define the *j*-th compartment), *N*_*j*_(*t*), is modelled by the following system of ODEs,

dN0dt(t)=−(α0+β0)N0(t),dNjdt(t)=2αj−1Nj−1(t)−(αj+βj)Nj(t),j=1,...,J.
 MathType@MTEF@5@5@+=feaafiart1ev1aaatCvAUfKttLearuWrP9MDH5MBPbIqV92AaeXatLxBI9gBaebbnrfifHhDYfgasaacH8akY=wiFfYdH8Gipec8Eeeu0xXdbba9frFj0=OqFfea0dXdd9vqai=hGuQ8kuc9pgc9s8qqaq=dirpe0xb9q8qiLsFr0=vr0=vr0dc8meaabaqaciaacaGaaeqabaqabeGadaaakeaafaqaaeGabaaabaWaaSaaaeaacqWGKbazcqWGobGtdaWgaaWcbaGaeGimaadabeaaaOqaaiabdsgaKjabdsha0baacqGGOaakcqWG0baDcqGGPaqkcqGH9aqpcqGHsislcqGGOaakiiGacqWFXoqydaWgaaWcbaGaeGimaadabeaakiabgUcaRiab=j7aInaaBaaaleaacqaIWaamaeqaaOGaeiykaKIaemOta40aaSbaaSqaaiabicdaWaqabaGccqGGOaakcqWG0baDcqGGPaqkcqGGSaalaeaafaqabeqacaaabaWaaSaaaeaacqWGKbazcqWGobGtdaWgaaWcbaGaemOAaOgabeaaaOqaaiabdsgaKjabdsha0baacqGGOaakcqWG0baDcqGGPaqkcqGH9aqpcqaIYaGmcqWFXoqydaWgaaWcbaGaemOAaOMaeyOeI0IaeGymaedabeaakiabd6eaonaaBaaaleaacqWGQbGAcqGHsislcqaIXaqmaeqaaOGaeiikaGIaemiDaqNaeiykaKIaeyOeI0IaeiikaGIae8xSde2aaSbaaSqaaiabdQgaQbqabaGccqGHRaWkcqWFYoGydaWgaaWcbaGaemOAaOgabeaakiabcMcaPiabd6eaonaaBaaaleaacqWGQbGAaeqaaOGaeiikaGIaemiDaqNaeiykaKIaeiilaWcabaGaemOAaOMaeyypa0JaeGymaeJaeiilaWIaeiOla4IaeiOla4IaeiOla4IaeiilaWIaemOsaOeaaiabc6caUaaaaaa@79C1@

The term 2*α*_*j *- 1_*N*_*j *- 1_(*t*) for *j *≥ 1 represents the cell birth (influx from the previous compartment because of division), whereas the term (*α*_*j *_+ *β*_*j*_)*N*_*j*_(*t*) represents cell loss (outflux from the compartment) due to division and death. The model (15) allows an analytical solution which was used in the parameter estimation procedure.

To estimate the best-fit parameters of this model, we used the objective function

Φ(p)=∑i=14∑j=0J(Nji−Nj(ti;p))2,
 MathType@MTEF@5@5@+=feaafiart1ev1aaatCvAUfKttLearuWrP9MDH5MBPbIqV92AaeXatLxBI9gBaebbnrfifHhDYfgasaacH8akY=wiFfYdH8Gipec8Eeeu0xXdbba9frFj0=OqFfea0dXdd9vqai=hGuQ8kuc9pgc9s8qqaq=dirpe0xb9q8qiLsFr0=vr0=vr0dc8meaabaqaciaacaGaaeqabaqabeGadaaakeaacqqHMoGrcqGGOaakieaacqWFWbaCcqGGPaqkcqGH9aqpdaaeWbqaamaaqahabaGaeiikaGIaemOta40aa0baaSqaaiabdQgaQbqaaiabdMgaPbaakiabgkHiTiabd6eaonaaBaaaleaacqWGQbGAaeqaaOGaeiikaGIaemiDaq3aaSbaaSqaaiabdMgaPbqabaGccqGG7aWoieqacqGFWbaCcqGGPaqkcqGGPaqkdaahaaWcbeqaaiabikdaYaaaaeaacqWGQbGAcqGH9aqpcqaIWaamaeaacqWGkbGsa0GaeyyeIuoaaSqaaiabdMgaPjabg2da9iabigdaXaqaaiabisda0aqdcqGHris5aOGaeiilaWcaaa@520E@

which corresponds, under a set of assumptions similar to those presented above for the PDE model, to the maximum likelihood approach, see [12] for details. Here Nji
 MathType@MTEF@5@5@+=feaafiart1ev1aaatCvAUfKttLearuWrP9MDH5MBPbIqV92AaeXatLxBI9gBaebbnrfifHhDYfgasaacH8akY=wiFfYdH8Gipec8Eeeu0xXdbba9frFj0=OqFfea0dXdd9vqai=hGuQ8kuc9pgc9s8qqaq=dirpe0xb9q8qiLsFr0=vr0=vr0dc8meaabaqaciaacaGaaeqabaqabeGadaaakeaacqWGobGtdaqhaaWcbaGaemOAaOgabaGaemyAaKgaaaaa@30B6@ and *N*_*j*_(*t*_*i*_; **p**) specify the data and the model solution for the cell generation *j *at time *t*_*i*_, **p **is the vector of estimated parameters *α*_*j *_and *β*_*j*_.

The best-fit parameter values *α*_*j *_and *β*_*j *_for data sets 1 and 2 are presented in Figs. 6 and 8. Note that these values are plotted at the middle of the intervals of the log10-transformed CFSE intensity which correspond to cells divided *j *times. Quantitatively, the rate functions *α*(*z*), *β*(*z*) and parameters *α*_*j*_, *β*_*j *_provide a different characterization of the cell kinetics. The qualitative bell-shaped behavior of the division rate with respect to the structure variable is similar for the PDE and ODE models (CFSE level and division number, respectively).

**Figure 9:**
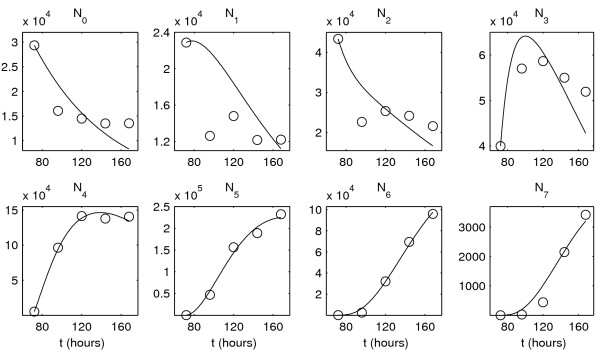
**Experimental data set 1 and the best-fit solution of the compartmental ODE model**. Experimental data are denoted by circles, the best-fit solution is denoted by solid lines. *N*_*j *_is the number of cells divided *j *times.

**Figure 10:**
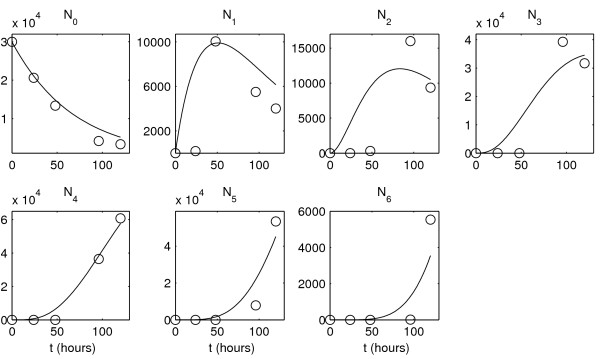
**Experimental data set 2 and the best-fit solution of the compartmental ODE model**. Experimental data are denoted by circles, the best-fit solution is denoted by solid lines. *N*_*j *_is the number of cells divided *j *times.

The ODE model provides a poorer fit of the total T cell population growth for data set 2, see Fig. 8 (right). In addition, this model fails to describe consistently the division number related structure of the cell populations which divided less than 3 times (except undivided cells for data set 2), as shown in Figs. 9 and 10. The above comparison suggests that the PDE model allows to describe in a more consistent way the dynamics of heterogenous CFSE-labelled cell populations and, therefore, reliably estimate the rates of the underlying turnover processes.

## Conclusion

Many immunological phenomena result from cell proliferation. To quantify the cell proliferation, the technology based upon flow cytometry in conjunction with fluorescent dye (such as CFSE) that stain cell membrane or cytoplasm is extensively used in experimental and clinical research. It provides large amounts of data on the evolution of the histograms of fluorescence intensity of the cell population growing in response to a perturbing agent. The challenge is not only to collect the data, but also to analyze them in a way that enhances our understanding of the kinetics of the cellular responses. To this end, mathematical models are needed that quantitatively describe and interpret the data, in particular allowing one to estimate the rates of cell division and death.

Recently a number of different mathematical approaches have been proposed for the analysis of CFSE data [7-10,12,17,25]. The corresponding models were instructive in appreciating the complexity of the parameter estimation problem from CFSE distribution data. These models take into account the heterogeneity of the growing cell populations with respect to the division number and are based upon systems of ordinary, delay or (age-structured) partial differential equations. None of these models considered the label intensity as a structure variable thus the CFSE histograms must be transformed into simplified descriptions of the generation structure of the population. This can be a vaguely defined procedure if the initial staining is not homogeneous. Such models, although easier to solve, cannot describe cell growth accurately enough due to the lack of structure information included.

In this study we developed a computational approach which allows a direct reference to the CFSE distributions. The label-structured cell population dynamics is described by a first order hyperbolic PDE model similar to those proposed by Bell and Anderson [19] for heterogenous cell populations structured by volume or size. The proposed model characterizes cell populations using the rate functions of cell division, death, label decay and the label dilution factor. We showed that this model provides a consistent mathematical tool for the analysis of CFSE-structured lymphocyte populations.

We presented a numerical approach for the parameter estimation implemented in the widely used package Matlab [38]. The major elements of this approach are: (*i*) the smoothing of the histograms of CFSE data, which generates a continuous functional approximation of the distribution density of the cell population with a reduced level of noise; (*ii*) the software for the solution of the initial-boundary value problem for the proposed PDE model using the second-order Lax-Wendroff scheme; (*iii*) the parameterization of the rate functions in order to reduce the variational problem of CFSE data assimilation to a finite-dimensional parameter optimization task; (*iv*) the maximum likelihood approach to the parameter estimation.

Two original data sets from in vitro CFSE proliferation assays with human T lymphocytes were used to evaluate the performance of the proposed approach. It was shown that the model quantitatively describes the kinetics of the cell populations both at the global level and with respect to the fine structure of the CFSE distribution. The estimated rate functions provide a deeper insight into the turnover kinetics of the growing T cell populations. By computing the mean values of the rate functions for consecutive CFSE ranges corresponding to the sequential generations, one can characterize the division number dependent T cell turnover rates. In particular, the estimated rate functions *α*(*z*) imply the following division number dependent proliferation rates [αj]j=07
 MathType@MTEF@5@5@+=feaafiart1ev1aaatCvAUfKttLearuWrP9MDH5MBPbIqV92AaeXatLxBI9gBaebbnrfifHhDYfgasaacH8akY=wiFfYdH8Gipec8Eeeu0xXdbba9frFj0=OqFfea0dXdd9vqai=hGuQ8kuc9pgc9s8qqaq=dirpe0xb9q8qiLsFr0=vr0=vr0dc8meaabaqaciaacaGaaeqabaqabeGadaaakeaacqGGBbWwiiGacqWFXoqydaWgaaWcbaGaemOAaOgabeaakiabc2faDnaaDaaaleaacqWGQbGAcqGH9aqpcqaIWaamaeaacqaI3aWnaaaaaa@36DF@ = [0.0023, 0.014, 0.020, 0.023, 0.010, 0.0032, 0.00002, 0] for data set 1 and [αj]j=05
 MathType@MTEF@5@5@+=feaafiart1ev1aaatCvAUfKttLearuWrP9MDH5MBPbIqV92AaeXatLxBI9gBaebbnrfifHhDYfgasaacH8akY=wiFfYdH8Gipec8Eeeu0xXdbba9frFj0=OqFfea0dXdd9vqai=hGuQ8kuc9pgc9s8qqaq=dirpe0xb9q8qiLsFr0=vr0=vr0dc8meaabaqaciaacaGaaeqabaqabeGadaaakeaacqGGBbWwiiGacqWFXoqydaWgaaWcbaGaemOAaOgabeaakiabc2faDnaaDaaaleaacqWGQbGAcqGH9aqpcqaIWaamaeaacqaI1aqnaaaaaa@36DB@ = [0.0033, 0.0088, 0.033, 0.016, 0.0082, 0.0011] for data set 2, where *j *stands for the division number. The best-fit estimate of the reduction factor for CFSE per cell after division is smaller than two, ranging from 1.9 (data set 1) to 1.7 (data set 2). A possible interpretation might be that the CFSE molecules bonded to proteins upon release from cells dying in the process of division can be taken up actively or adhere to the live cells. Interestingly, the a quantitative CFSE data analyses published recently also indicates that the factor difference in median fluorescence intensity of adjacent CFSE peaks is typically not exactly 2 [39] and there might be a few percent difference among siblings in the CFSE fluorescence inherited from the mother cell [24].

A number of issues require further systematic analysis: (*i*) the statistical error model underlying the fluctuations in the CFSE histograms; (*ii*) the level of noise smoothing used in the generation of the continuous distributions from the histogram data; (*iii*) the convergence of the finite-dimensional approximation of the rate functions estimation problem; (*iv*) the analysis of the confidence bounds for the estimated rate functions; (*v*) the application of Tikhonov regularization for the function identification (inverse) problem.

Overall, our study suggests that the label-structured modelling of cell population balance could become a component of the CFSE flow cytometry analysis software. The model's modifications can be used as building blocks for integrative mathematical description of complex in vivo labelling experiments in infected subjects such as those presented recently in [40,41] for investigation of T cell activation and homeostasis.

## Competing interests

The author(s) declare that they have no competing interests.

## Authors' contributions

TL performed the numerical identification work and drafted together with DR and GB the manuscript. DR supervised the computations with PDEs. TS and MS conducted the CFSE proliferation experiments on CD3/CD28 antibody stimulation. SE designed experimental study that provided the data set on PHA stimulation. AM performed the experimental design for data set on CD3/CD28 antibody stimulation. GB conceived the study and participated in its design and co-oordination. All authors have read and approved the final manuscript.
